# Consumer Health Information Technology in the Prevention of Substance Abuse: Scoping Review

**DOI:** 10.2196/11297

**Published:** 2019-01-30

**Authors:** Apoorva Milind Pradhan, Leah Park, Fadia T Shaya, Joseph Finkelstein

**Affiliations:** 1 Department of Health Services Research University of Maryland School of Pharmacy Baltimore, MD United States; 2 Icahn School of Medicine at Mount Sinai New York, NY United States

**Keywords:** consumer health information technology, primary prevention, substance abuse, review

## Abstract

**Background:**

Addiction is one of the most rapidly growing epidemics that currently plagues nations around the world. In the United States, it has cost the government more than US $700 billion a year in terms of health care and other associated costs and is also associated with serious social, physical, and mental consequences. Increasing efforts have been made to tackle this issue at different levels, from primary prevention to rehabilitation across the globe. With the use of digital technology rapidly increasing, an effort to leverage the consumer health information technologies (CHITs) to combat the rising substance abuse epidemic has been underway. CHITs are identified as patient-focused technological platforms aimed to improve patient engagement in health care and aid them in navigating the complex health care system.

**Objective:**

This review aimed to provide a holistic and overarching view of the breadth of research on primary prevention of substance abuse using CHIT conducted over nearly past five decades. It also aimed to map out the changing landscape of CHIT over this period.

**Methods:**

We conducted a scoping review using the Arksey and O’Malley’s modified methodological framework. We searched 4 electronic databases (PubMed, Cochrane, Scopus, and EMBASE). Papers were included if the studies addressed the use of CHIT for primary prevention of substance abuse and were published in English between 1809 and 2018. Studies that did not focus solely on primary prevention or assessed additional comorbid conditions were eliminated.

**Results:**

Forty-two papers that met our inclusion criteria were included in the review. These studies were published between 1970 and 2018 and were not restricted by geography, age, race, or sex. The review mapped studies using the most commonly used CHIT platforms for substance abuse prevention from mass media in the 1970s to mobile and social media in 2018. Moreover, 191 studies that were exclusively focused on alcohol prevention were excluded and will be addressed in a separate paper. The studies included had diverse research designs although the majority were randomized controlled trials (RCT) or review papers. Many of the RCTs used interventions based on different behavioral theories such as family interactions, social cognitive theories, and harm-minimization framework.

**Conclusions:**

This review found CHIT platforms to be efficacious and cost-effective in the real-world settings. We also observed a gradual shift in the types and use of CHIT platforms over the past few decades and mapped out their progression. In addition, the review detected a shift in consumer preferences and behaviors from face-to-face interactions to technology-based platforms. However, the studies included in this review only focused on the aspect of primary prevention. Future reviews could assess the effectiveness of platforms for secondary prevention and for prevention of substance abuse among comorbid populations.

## Introduction

### Background

Addiction has been identified as the most neglected disease in the United States, with nearly 40 million Americans over the age of 12 years meeting the clinical criteria for addiction involving nicotine, alcohol, or other drugs [1]. In addition, it is also estimated that nearly 80 million people in the country are *risky substance users*, meaning that although they are not addicted, they use tobacco, alcohol, and other drugs in ways that could be harmful and threaten public health and safety [1]. Thus, addiction has been established as a growing epidemic. In 2017, the American government spent in excess of US $740 billion in costs related to crime, lost work, and health care, and costs are growing exponentially [2-6].

However, the burden of drug and alcohol use and abuse is not just limited to the United States. The World Health Organization (WHO) estimates the global burden of disease related to drug and alcohol abuse to be nearly 5.4% [3]. Drug use is associated with grave long-term and short-term health implications and is recognized as one of the avoidable causes of mortality [7]. According to the National Institute on Drug Abuse, in the United States, nearly 64,070 people died of drug overdose in 2016, and this number is rapidly increasing [8]. Given the dire medical, social, and economic consequences associated with increasing drug use, the governments and institutions around the world have been working tirelessly to develop strategies to combat this drug epidemic.

Among the many interventions developed to address the drug epidemic, prevention strategies have shown to be effective to reduce the onset of drug use and risk of drug abuse [9]. Prevention in the field of addiction and substance abuse may be classified as primary, secondary, and tertiary [10]. Primary prevention could be defined as efforts to improve health and forestall the onset of substance abuse and delay the first use (WHO). Primary prevention can be targeted toward the general population, individuals who are at risk of substance abuse, or those who have signs indicating predisposition to developing addiction [11]. As substance abuse can lead to other medical and social problems and have a negative impact not only on individuals, their families, and the society (Substance Abuse and Mental Health Services Administration), effective primary prevention strategies can have beneficial cascading effects in the long run [12].

Among the various strategies implemented for prevention, consumer health information technology (CHIT) has emerged as a potentially effective way to prevent and treat substance abuse [13]. CHIT has been defined in several ways [14-18]. For the purpose of our review, we decided to adopt a combination of 2 definitions of CHIT by Or et al and Tao et al to broaden our scope of work [14,17,18]. Or et al have defined CHIT as “patient-focused interactive web- or technology-mediated applications that are designed to improve information access and exchange, enhance decision making, provide social and emotional support, and facilitate behavior changes that promote health and wellbeing” [14,18], whereas Tao et al have defined it as “consumer-centered electronic tools, technologies, apps, or systems that are interacted with directly by health consumers to provide them with data, information, recommendations, or services for promotion of health and health care” [17]. CHIT has experienced an exponential growth during the recent decades [13]. The traditional CHIT platforms for health promotion such as educational audio and video materials, along with the rapid proliferation of new modalities, take advantage of the wide accessibility of health-related content via internet, mobile phones, and social media [19]. By 2016, nearly 88% Americans were using the internet [20], 92% a cell phone, and 76% social media [21]. Given the reach of these modalities, technologies such as the internet and mobile phones are viewed as a promising platform for affecting assessment, prevention, treatment, and recovery of substance abuse disorders by national and global organizations [22].

In the past few decades, multiple research studies have been conducted on the use of CHIT in the prevention of substance abuse, but the reflective step of looking broadly across this vast research corpus is yet to be undertaken. Previous reviews on CHIT and substance abuse have assessed the impact of CHIT with narrow focuses in terms of targeted study populations and specific types of technology [23-25]. Moreover, 1 systematic review evaluated benefits, potentials, and shortcomings of recent technology such as social media and mobile apps as an intervention for substance use among those who also have HIV. This study concluded the new technology is well accepted and has good feasibility with great potential for educating people on sensitive topics [25]. Another study investigated the benefits of technology on prevention and treatment of substance use among young people and reported that technology is particularly effective in both prevention and treatment regardless of the stage of substance abuse [23]. Moore et al reported that the use of computers in the prevention of drug abuse was effective in reducing substance use and improving knowledge, leading to greater motivation to change behavior and was well accepted by users [24].

Although these reviews presented the impact of CHIT on substance abuse from various perspectives, there has not been a comprehensive review of how interventions using CHIT have shaped primary prevention of substance abuse over the years. Furthermore, some of the previous reviews were limited to studies that used technologies used in recent decades [23,25]. Before the advent of mobile phones and internet, other types of technologies, including phone, television, radios, and videos, played a prominent role in prevention of substance abuse [26-30]. Although some of these technologies from the previous decades are no longer in widespread use, there are valuable lessons that can be learned from which past strategies using technology-based interventions were effective or ineffective and how technology has evolved over the years. Due to the lack of studies that provided overall assessment of effectiveness of CHIT on primary prevention of substance abuse, we focused the scope of our review on primary prevention of various types of substance abuse and identified studies that provided prevention efforts to reduce the new onset of substance abuse.

### Objective

In this study, we present a scoping review of the breadth of research over the past few decades, specifically with the use of CHIT in the primary prevention of substance abuse. The objective of this review was to describe the use of CHIT in the primary prevention of substance abuse over the last five decades and examine the changes and developments in the types of CHIT employed for this effort. Our goal was also to summarize these preventive approaches and report lessons learned from these studies.

## Methods

After considering the multiple systematic approaches that are used for the review of published literature, we decided to undertake a scoping review to map out the changing trends in the use of CHIT in the substance abuse prevention landscape over the past few decades. The scoping review methodology is more commonly known as mapping, a process of summarizing the range of evidence to convey the depth and the breadth of the published literature in a particular field of interest. Unlike systematic reviews and meta-analysis, scoping reviews are neither limited by the type of study under consideration nor do they evaluate their quality. Yet, it enables the researcher to examine the extent, range, and nature of research activity; determine whether a full systematic review would be of value; summarize and disseminate the research findings; and identify gaps in the literature [31-34].

In designing our scoping review, we used Arksey and O’Malley’s pioneering framework and incorporated recent scoping review publications as well. Arksey and O’Malley’s scoping review framework outlines a 5-stage approach, which was further adapted and modified to some extent by others to develop a more feasible approach for reviewing such a vast body of literature [31,32]. The 5 steps are each discussed below.

### Identifying the Research Questions

The growing drug abuse epidemic in the United States underscores the need for exploring new approaches to prevention. The ubiquitous nature of CHIT in our day-to-day lives presents an opportunity to study its potential as a tool for substance abuse prevention. Our intent, thus, was to learn the extent of the present use of CHIT platforms in substance abuse prevention; however, the scope of this review was only limited to primary prevention, and not secondary prevention. In addition, we intended to explore the best methods to leverage CHIT platforms in the future among high-risk individuals for primary prevention. Our goal was to examine the following: (1) extent, range, and nature of the evidence; (2) identify gaps in the literature; and (3) summarize and disseminate this information to guide practice and policy. Following Levac et al’s suggestion to enhance and advance Arksey and O’Malley framework, our team clarified and linked the purpose and the research question from the beginning of this study. To avoid leading with a *highly focused research question*, we asked a sufficiently broad question: what is the role and scope of CHIT in the primary prevention of substance abuse? [31,32]. To further guide our review, we formulated 4 subquestions:

1. What are the demographics related to substance abuse disorder studied?
  - Locations—study site—the United States, the United Kingdom, and multicountry
  - Demographics of the study population and sample size of the study
  - Length of observation—long-term impact versus short-term impact
2. What is the type of intervention and the behavioral framework, if any, used?
  - Intervention—primary prevention
  - Behavioral framework—transtheoretical model, motivational interviewing, brief intervention, acceptance and commitment therapy, and psychoeducation
3. What is the type of CHIT used?
  - CHIT—desktop, tablet, mobile phone, internet, interactive voice response, video or movies, video recording or audio recording, and radio
  - Social media—Baidu Tieba, Facebook (and its associated Facebook Messenger), Gab, Google+, Myspace, Instagram, LinkedIn, Pinterest, Tumblr, Twitter, Viber, VK, WeChat, Weibo, WhatsApp, Wikia, Snapchat, and YouTube
4. What are the major takeaways from the literature in terms of outcomes and is the intervention effectiveness evident within the literature?
  - Outcomes—change in knowledge, attitudes, and behaviors
  - Effectiveness—intervention outcomes presented by the author(s) and their suggestions for future research

### Identifying Relevant Studies

Arksey and O’Malley in their study had emphasized the need to be comprehensive while conducting a scoping review [31]. At this stage, our team deliberated and decided on the various search terms, databases, search strategies, and eligibility criteria. To maintain a broad approach, we did not limit the inclusion of studies by the type of substance used. With the aid of a librarian, we searched electronic databases such as PubMed, Scopus, EMBASE, and the Cochrane library. Various search terms and their combinations were used to identify relevant studies, which discussed the use of CHIT in substance abuse prevention: “technology, internet, cell phone, multimedia, computer-assisted, telemedicine, social media, internet, web-based, etc. with prevention and control, preventive health services and substance-related disorders, substance abuse, substance misuse, drug addiction.” An exhaustive list of the search terms used can be found in the Multimedia Appendix 1.

### Eligibility Criteria

The following inclusion criteria were used to guide the search and were also used for reviewing papers:

- Publication language—English
- Only limited to human subjects
- Time range—from 1809 to January 2018
- All age groups
- Review papers included—research studies, systematic reviews, meta-analysis, narrative reviews, observational studies, randomized control trials (RCT), qualitative studies, completed clinical trials, and dissertations and working papers
- Reviews, including but not limited to developed countries, given the growing drug abuse presence all around the world. Studies were included from the United States, the United Kingdom, Canada, Europe, Middle East, South America, Southeast Asia, New Zealand, and Australia
- Studies that address the role of CHIT in primary prevention of only substance abuse—defined as strategies to prevent initiation of substance abuse

Exclusion criteria are as follows:

- Journal papers that are not research studies or reviews (ie, those besides the ones defined in the inclusion list) such as editorial reviews, commentaries, opinion papers, and book reviews
- Research targeting secondary prevention strategies such as treatment, maintenance, relapse, and interventions
- Research studies that lacked the use of CHIT as a part of their interventions for primary prevention
- Research studies aimed at prevention of substance abuse among individuals with comorbidities such as HIV and risky health behaviors
- Research studies conducted in special populations such as cancer patients and patients with HIV, AIDS, or other sexually transmitted diseases (STDs)

## Results

### Study Selection

The literature search yielded 4393 papers. Following the search, the study selection was conducted in 2 parts. First, a single reviewer conducted a title screening process based on the inclusion and exclusion criteria. At this stage of screening, any ambiguity after reviewing the title, with regard to the context of the study, did not eliminate it from being considered for the next step. After completion of this step, 1606 papers were identified; then based on the eligibility criteria, 2 reviewers independently conducted abstract coding. On completion of abstract coding, the inclusion of the paper for full-text review was determined by corroboration from both the reviewers. Given the objective of this review, we wanted to assess the overall breadth of the literature on alcohol; thus, our initial yield for all studies included alcohol whether with or without other substances. On the initial screen, we only kept those studies that considered alcohol along with other substances to make sure that we address the impact of CHIT in prevention of all types of substance abuse without our conclusions being skewed because of any single substance of abuse. We think that it is important to note that about 79.2% (191/241) of all the overall CHIT literature on substance use addressed only alcohol, whereas 20.7% (50/241) addressed other substances with or without alcohol. Our intent was to include studies addressing alcohol to the extent that they also addressed the concurrent use of other substance. Given the epidemiology and suggestive evidence of alcohol being a gateway to other drugs, we decided to separately synthesize the studies focused singularly on alcohol in an independent review altogether. Hence, for the purpose of this review, papers focusing solely on alcohol abuse prevention were excluded. We only included studies that focused solely on substance abuse prevention. Studies that looked at substance abuse prevention in conjunction with other morbidities such as prevention of HIV, or other areas of education such as undertaking risky sexual behaviors, were excluded from this review.

**Figure 1:**
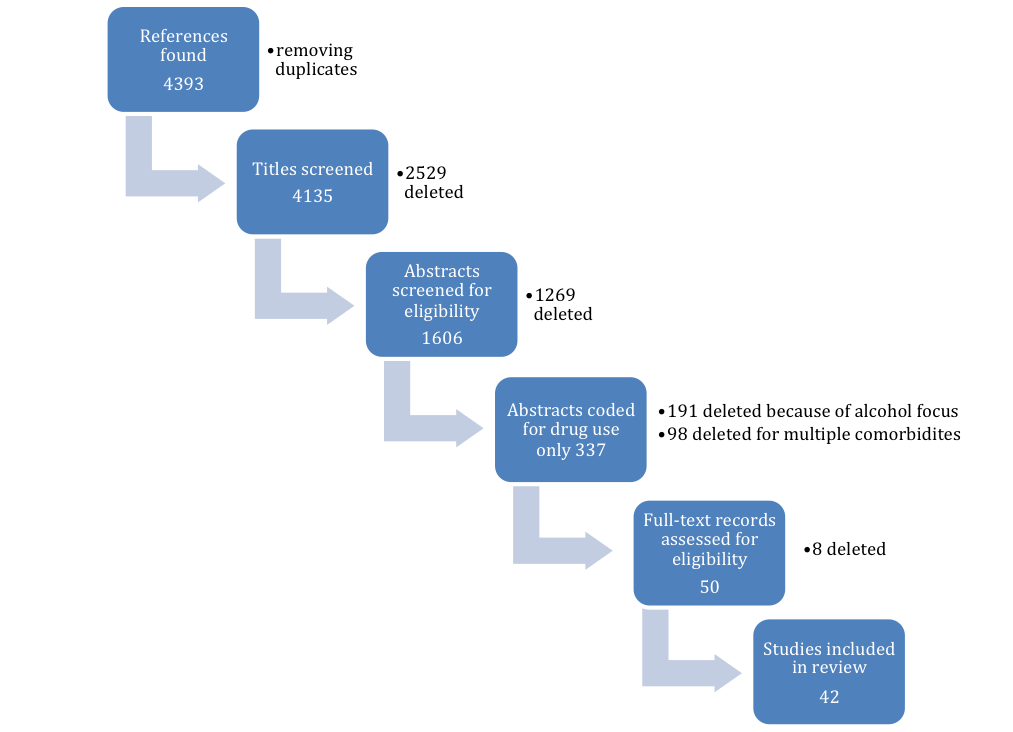
Preferred Reporting Items for Systematic Reviews and Meta-Analyses flowchart for literature search process and inclusion.

Following abstract coding, 50 papers were included for a full-text review. At the end of the full-text review, 42 papers were found to meet all our inclusion criteria and were included in the study. The specific steps of study selection and the number of papers included and excluded in each step can be seen in Figure 1 (flowchart for literature search and inclusion in Multimedia Appendix 2). After the study selection, information relevant to each of the main review questions was extracted and analyzed. We developed a standardized table (Multimedia Appendix 2) using these 42 papers selected to assess the different forms of CHIT used and their impact on the prevention of substance use. We also used this table to identify the different underlying behavioral frameworks most commonly at play in these interventions. Additionally, we also created a table to compile all the papers included in the reviews that are enlisted in this scoping review (Multimedia Appendix 3 [35-205]).

### Locations

Most studies included in the review were conducted in the United States. Countries other than the United States were Canada [206-208], Australia [207-212], Brazil [213], the Netherlands [214], New Zealand [29], Norway [209,215,216], the United Kingdom [28,209,217], Germany [207], and Switzerland [218]. Moreover, 1 systematic review included 7 studies from the United States, and 1 from Norway [216]. Another study reviewed trials that took place in Australia, the Netherlands, the United Kingdom, and the United States [209]. In addition, 1 RCT took place in the United States and Canada [206]. A total of 6 studies included in the review were conducted in Australia. Within the United States, 1 randomized study recruited participants in West Virginia and Ohio [219]. Another RCT in the United States chose participants from 19 states and included Asian populations [220]. Furthermore, 1 study using Monitoring the Future survey data included nationally representative sample of students from 48 states [221]. Studies from other states in the United States recruited participants from California [30,222], New York [223], New Jersey [223], Connecticut [223], Texas [224], Kansas [222], Michigan [225], Missouri [222], and South Carolina [27]. Moreover, 1 study reported having participants mostly from rural communities in South Carolina [27]. Another study chose participants from schools in a semirural community in Michigan [225].

### Participants

We did not exclude any studies in the review based on the demographic characteristics of participants. Therefore, study participants varied in ages, racial and ethnic backgrounds, and socioeconomic status. A total of 13 studies assessed school- or college-based intervention programs, and thus, participants were students, students with parents, or teachers [26,30,210,213,217,221,222,224-229]. Moreover, 1 quasi-experimental study included participants enrolled in vocational schools [218]. Similarly, 30 studies included only young people, adolescents, or school-aged children from 11 to 24 years of age. However, some systematic reviews assessed studies across all ages, both children and adults [207,230-233]. Some studies included only racial and ethnic minorities such as Asians [220], Hispanics [223], and African Americans [223]. Although some studies did not specify racial background of participants, 2 studies reported having study subjects from various races, including white, African American, Hispanic, and Asian [30,224]. Moreover, 1 study in the United Kingdom having subjects from 7 schools included 1 school with predominantly black students and the rest with mostly white students [217]. Another UK school-based study reported their participants were chosen to have a balance between both sexes, residence in rural and urban areas, and varying intellectual abilities [28]. A total of 4 studies specifically evaluated interventions on adolescent girls [220,223,234,235]. Of the 4 studies, 3 studies used a mother-daughter prevention approach [220,223,227], assessing the impact of programs both on adolescent girls and their mothers. Moreover, 1 study selected participants only from economically disadvantaged African American adolescents [236].

### Sample Size

A range of study sample size was included in the review. Overall, 1 systematic review had a total of 52,746 individuals as participants from 8 studies [215]. Other reviews included study sample sizes as small as 38 [207] to as large as 8352 [209]. Mother and daughter programs ranged from sample sizes of 206 to 916 girls and their mothers [220,223,234]. The nationally representative survey data included 337,918 cases [221]. Quasi-experimental studies also included both small (n=26) and large (n=2882) sample sizes [30,236]. The RCTs reported larger sample sizes in general, ranging from 179 to 2332 participants [229].

### Comparators

Of the 42 studies included in this review, Table 1 shows the different CHIT platforms used by the studies included. Moreover, 4 studies (Schuman et al, 1971; Milne et al, 1975; Pickens, 1984; and Eiser et al, 1988) looked at the effects of films as a part of educational programs to promote discussion around the areas of substance use [28,29,217,225]. The Schuman et al’s (1971) study was a cross-sectional study conducted to evaluate the results of a field test implemented to emphasize on aspects such as motivations governing drug behaviors as opposed to drug facts [225]. The Milne et al’s (1975) study used a pre-post study design [28], whereas the Eiser et al’s (1988) study used an RCT design to assess the effectiveness of films as a medium of drug education [217]. The Pickens’ (1984) study was a literature review aimed at assessing the effectiveness of films in drug education compared with other forms of media [29].

A total of 10 studies looked at the use of different mass media interventions in general. Of these, 7 studies (Wallack, 1980; Wallack, 1981; Bandy et al, 1983; Flay et al, 1983; Flay et al, 1986; Brinn et al, 2010; and Carson et al, 2017) were literature reviews, which evaluated the use and effectiveness of mass media as a tool for substance abuse prevention [215,216,226,231,237-239]. The Barcus et al (1975) and the Kinder’s (1975) studies examined the impact of mass media on attitudes associated with increased substance use [230,240]. Although majority of the studies evaluated the impact of mass media on multiple drug use, the Brinn et al (2010) and the Carson et al’s (2017) studies looked specifically into its role in smoking prevention [215,216]. The Miller et al’s (1981) study used a cross-sectional survey design to compare effectiveness of media platforms such as television, radio, and newspaper in dissemination of substance abuse education [27]. Moreover, 5 studies looked specifically into the role of television and radio as modes of interventions. In addition, 3 studies (Feingold et al, 1977; Sussman et al, 1987; and Brannon et al, 1989) used a quasi-experimental design [26,30,222]. The Sussman’s study and the Brannon’s study used a school-based television program format [30,222], whereas Feingold (1977) and Terry-McElrath et al (2011) used television advertisements as their mode of intervention [26,221]. The Johnson et al’s (1989) study was the only one that reviewed the strategies and research efforts in the use of radio and television [232].

Post 2009, there was a notable surge in the number of computer- and internet-based interventions and a subsequent decrease in the number of mass media–based interventions. There were in total 18 studies that used Web-based and internet-based programs for interventions, whereas 5 studies used the desktop-enabled software or CD-ROM-based programs for interventions. Moreover, 13 of these studies used an RCT design and aimed to evaluate the effectiveness of computer-based substance abuse prevention programs. The Hansen et al, Newton et al (2014 and 2016), Christoff Ade et al (2015), and Andrews et al (2011 and 2014) studies evaluated the comparative effectiveness of computer-delivered prevention or screening programs with those termed as usual or traditional models of delivery [210-213,228,229,241]. In addition, 4 studies performed by Schinke et al compared the effectiveness of a mother-daughter–based program with a control group involving no intervention [220,223,227,234]. Furthermore, 2 studies by Schwinn et al tested the effect of an internet-based gender-specific drug prevention program between girls with and without an intervention [206,235]. Another 2 studies used the quasi-experimental pre-post study design, the Klisch et al’s (2013) study compared the effectiveness between 2 different Web-based interactive programs [224], and the Moncher et al (1989) assessed the efficacy of the computer-delivered prevention programs [236]. Of the remaining studies, 5 studies were systematic reviews aimed at expanding the base of research and synthesizing the effectiveness of computer- and Web-based prevention programs.

The review by Carson et al (2017) and the randomized trial by Schwinn et al (2017) specifically included the effectiveness of social media as a component of the prevention programs [216,235]. In addition, a quasi-experimental (pre-post assessment) study by Haug et al (2017) and 2 systematic reviews by Jiang et al (2017) and Kazemi et al (2017) evaluated and critiqued the effectiveness of mobile phone–based prevention programs [218,233,242].

**Table 1:** Technology used as intervention in the 42 studies reviewed

Technology	Studies, n (%)
Computer	6 (10)
CD ROM	5 (9)
Film	4 (6)
Internet	18 (31)
Mass media	6 (10)
Mobile	3 (5)
Radio	6 (10)
Television	11 (19)

### Length of Observation

Of the 42 studies reviewed here, 17 studies were in the form of systematic reviews spanning over the past 60 years. Of the remaining 27 studies, 8 studies had a short-term follow-up period of 1 month or less (Schuman et al; Milne et al, 1975; Feingold et al, 1977; Eiser et al, 1988; Moncher et al, 1989; Andrews et al, 2011; Deitz et al, 2011; and Klisch et al, 2013) [26,28,217,219,224,225,236,241]. Most of these studies used a cross-sectional pre-post assessment format. The long-term follow-up periods for most of the remaining studies ranged between 6 months (Schwinn et al, 2010; Champion et al, 2016; and Haug et al, 2017) to 1 year (Sussman et al, 1987; Schinke et al, 2009; Fang et al, 2010; Newton et al, 2014; Newton et al, 2016; and Schwinn et al, 2017) [30,206,210,212,218,220, 234,235]. In addition, 2 studies, Schinke et al’s (2009) that evaluated computer-delivered program in preventing abuse among adolescent girls and Andrews et al’s (2014) that assessed the long-term efficacy of a tobacco prevention program, had a follow-up period of 2 years [227,229]. The study by Christoff Ade et al in 2015 compared the efficacy of 3 different interventions, including a computer-delivered one, and followed its participants for 3 months [213]. The longest study period in this review was 10 years (Terry-McElrath et al, 2011); it evaluated the impact of antidrug advertisement exposure and campaign-specific exposure on the attitudes, beliefs, and behaviors among youths from 1995 to 2006 [221].

### Outcomes and Results

Of the 4 studies that looked at films as the mode of intervention delivery, the Schuman et al’s (1971) study found no significant difference in the identification of drug clues by geographical or socioeconomic differences [225]. It also found a large gap in perceptions about drugs among faculty and students [225]. The Milne et al’s (1975) study found no significant difference in knowledge and attitudes toward drug use. Instead, results showed that students who believed drug use had social advantages also held onto the concept that the dangers of drug abuse were over exaggerated, a finding that thereby emphasizes the need for drug education [28]. The review study conducted by Pickens et al (1984) did not find film interventions superior to nonfilm approaches and found that the short-term impact of film interventions did not last in long-term follow-up studies [29]. However, the Eiser et al’s (1988) study showed that an entertaining drug prevention film might be more effective in leading students to reject dangerous substances. In contrast, the students who viewed the educational film regarded both illegal and legal drugs to be similarly dangerous and addictive [217].

Studies that used mass media, radio, and television as modes of intervention found that neither of these platforms as stand-alone were adequate to bring about a change in the overall attitudes and behaviors of people who engage in substance use. The Barcus et al (1975), Wallack et al (1980), Wallack et al (1981), and Flay et al’s (1983) studies found that mass media alone is not sufficient to affect behavioral changes and that it needs to be supplemented by school- or community-based prevention programs [215,216,230,231,237-240]. In addition, the literature reviews conducted on the use of mass media by Kinder (1975), Bandy et al (1983), Brinn et al (2010), Carson et al (2017), and a study by Sussman et al (1987) found either inconclusive or conflicting end results pertaining to the use of mass media in disseminating drug-related information and bringing about attitude changes [30,215,216,230,231]. Some studies (eg, Feingold et al, 1977) also found a boomerang effect of the use of television and radio, and antidrug messages were found to potentially lead to drug use [26]. Another exploratory study by Miller et al (1981) evaluated the comparative effectiveness among different mass media platforms such as television, radio, and newspaper and found that it depended on the demographics of the target audience: the results varied by race, sex, and geographical area [27]. Only the Brannon et al’s study (1989) specifically looked at the effectiveness of television as a delivery format found it to have higher classroom participation rates, greater satisfaction, and higher perceived effectiveness for a combined television and classroom program, thus concluding it to be a viable option for wider implementation [222].

Post 2009, there was a notable increase in the number of studies that used computer- and Web-based interventions for substance use prevention. A total of 4 studies conducted by Schinke et al between 2009 and 2011 used mother-daughter dyads from different races to study the effectiveness of computer-delivered interventions based on the family interaction theory [220,223,227,234]. All studies found significant reductions in risk factors, drug use, and an increase in the protective factors. Some studies also showed improvements in the quality of mother-daughter relationships. Moreover, 2 studies conducted by Schwinn et al in 2010 and 2017 used gender-specific interventions for girls using internet and social media platforms. These studies found reduced 30-day alcohol, marijuana, poly-drug, and total substance use at 6-month and 1-year follow-up [206,235]. The 2017’s study also found material changes in the cognition and skills that are empirically linked to drug use risks [235]. In addition, 2 studies by Andrew et al in 2011 and 2014 analyzed the short- and long-term efficacy of *Click City tobacco intervention* and found that the intervention had the potential to significantly postpone or prevent the initiation of cigarette use and regular smoking among students. In addition, although in the short term, the intervention showed moderate effectiveness at changing intentions to use smokeless tobacco in the future, the effect did not persist in the long term [229,241]. Another study (Deitz et al, 2011) that evaluated the effect of the Smart Rx Web program found that it significantly increased participants’ knowledge of proper prescription drug use and improved their self-efficacy in ability to manage and adhere to appropriate treatments [219].

Multiple studies identified in this review had used school-based programs for the delivery of Web-based interventions; 3 of these conducted by Newton et al between 2013 and 2016 in Australia used the climate schools format for the prevention of use of drugs such as cannabis, alcohol, ecstasy, and new psychoactive substances (NPS) [210-212]. These studies not only found evidence that internet-based preventive interventions significantly decreased substance use but also demonstrated that they could concurrently reduce associated risk factors in adolescents. However, the intervention neither significantly changed binge drinking and cannabis nor ecstasy and NPS use in the short term; the effects of these interventions were only apparent after 12 months, thereby showing a time-delayed effect, which could be attributed to the time required by the students to experience and implement the strategies learned [212]. The Hansen et al’s study in 2009 that evaluated the efficacy of Web-based components to facilitate program implementation concluded that school-based prevention programs could benefit from adding Web-based components to improve ease of implementation and enthusiasm of teachers [228]. The Klisch et al’s (2013) study, which used interactive game sessions in 11th and 12th graders, found the intervention to be effective in promoting healthier attitudes toward nonmedical use of prescription drugs [224]. Similarly, the Haug study in 2017, which used a mobile phone–based intervention found that it improved study participation, retention, and improved effectiveness with a statistically significant increase in the life skills and self-management skills and reduction in the number at risk for alcohol use [218]. However, a study conducted by Christoff Ade et al in 2015 could not find conclusive evidence of effectiveness of computer-based intervention among college students for reducing substance use [213].

A total of 4 systematic reviews conducted by Champion et al (2013 and 2016), Wood et al (2014), and Hopson et al (2015) on the use of computer- and internet-based programs found them to be potentially efficacious methods of delivering drug prevention programs. The Champion et al’s reviews found greatest effects in relation to drug- and alcohol-related knowledge with persisting effectiveness at 6- and 12-month follow-ups [209,214]. The Wood et al’s review, on the other hand, emphasized the need for further research to better understand the value of human contact in health interventions and to determine the optimal levels of professional input [207], whereas the Hopson et al’s (2015) review identified computer- and internet-based programs as cost-effective options for reaching more individuals, but on the whole reported mixed findings in terms of the effectiveness over traditional methods [208]. In addition, 2 reviews conducted by Jiang et al (2017) and Kazemi et al (2017) reviewed the use of telephone and mobile technology in substance use prevention and found that although it was a promising means to address substance use, the studies included in the reviews for the most part showed either inconclusive or mixed results in terms of the efficiency and efficacy [233,242].

## Discussion

### Principal Findings

This review included studies spanning across the globe, with the target population for these studies ranging across varying age groups, race or ethnicity, and gender and having differing study designs and sample sizes. The period for the literature search ranged from 1809 to January 2018, but the search only captured studies going as back as the early 1950s. This could be attributed to the fact that the review only included studies that had digitalized records enlisted on the databases searched. However, it can be said with fair amount of certainty that this review manages to capture majority of the trends in the use of CHIT. The boom in the use of media and CHIT platforms was seen to have occurred post the Second World War, thereby reaffirming the validity of the literature search timeline [243].

This review explored multiple CHIT platforms such as television, radio, films, mass media, computer, CD-ROM, internet, social media, and mobile. It was observed that from 1971 to 1989, film, televison, radio, and mass media were the most commonly used modes of intervention [26-30,217,222,225,226,230-232,236-240], whereas post 2009, there was a greater emphasis on the use of computer- and internet-based interventions [206-215,219-221,223,224,227-229, 234,241]. Furthermore, recent years show a growing emphasis toward exploring the role of social media– and mobile phone–based interventions to expand the reach of these prevention programs [216,218,233,235,242].

CHIT-based interventions have been shown to overcome challenges imposed by in-person–delivered intervention strategies such as the need for trained personnel to prepare and deliver intervention programs [218]. In addition, studies that examined the impact of electronic health and mobile health (mHealth) interventions found them to be efficacious and cost-effective, with computer-based interventions being more cost-effective than other preventive measures that are labor intensive and costly such as Life Skills Training Program [244]. In our study, the use of media and technology to prevent substance abuse was also found to have several advantages as a prevention strategy. Technology-based interventions can facilitate rapid dissemination of information and improving knowledge about substance use [220,236,238,240]. They can also reduce intervention variability that may occur with a person-based intervention method [226], improving integrity of intervention measures. In addition, intervention recipients, especially nonabusers who are ideal candidates for primary prevention, are more likely to depend on the media to gain information and knowledge about substance abuse [228].

Studies based on the family interaction theory and aimed at improving relationship quality among mother-daughter duos and studies that were gender-based were found to be effective in reducing the substance use in both the long and short term [220,223,227,234,235]. Multiple studies included in the review used school-based programs for delivery of Web-based interventions; of these, the climate school studies conducted in Australia not only found evidence that internet-based preventive interventions significantly decreased substance use but also demonstrated that they can concurrently reduce associated risk factors in adolescents [209-212,214]. However, the intervention did not show significant impact in the short-term use of substances; the effects of these interventions were only apparent after 12 months, thereby showing a time-delayed effect, attributed to the time required by the students to experience and implement strategies learnt [209,211,212]. Other studies that explored the effectiveness of school-based programs using Web-based or mobile phone–based interventions also found similar results. The studies that focused on the use of social media and mHealth platforms suggested the growth of research and literature in this domain [216,218,233,235,242].

However, in this study, we also found that technology-based interventions are not a panacea in the prevention of substance abuse. Despite the great number of resources poured into development and implementation of media- and technology-based interventions, earlier and recent studies demonstrated moderate effectiveness of these strategies in changing attitude, and ultimately, behavior of recipients of interventions [210,228,235-239]. Although in cases where people may gain greater and more accurate knowledge in substance abuse and negative consequences ensuing from the use, studies failed to show the changes in terms of decreasing or terminating the use or abuse of substance because of these interventions [228,236,237]. For example, studies that explored the role of film, television, radio, and mass media did not find any conclusive evidence to support the stand-alone effectiveness of these platforms. Most of the studies concluded that these platforms should be used in conjunction with other prevention initiatives [26,28,30,222,225,226,237-240]. 

This review also shows a gradual shift in the types and use of CHIT platforms over the past few decades. It has slowly moved from mass media–based interventions toward Web-based interventions, and following the current trends is heading toward a greater emphasis on telehealth and mHealth-based interventions. We live in an age in which most people frequently use technology and social media and are acutely aware of the current opioid misuse and substance abuse predicament facing by the United States in general and the world at large [22,245]. In this social context, technology could be useful to reach the general population as well as specific at-risk population and potentially be used to develop more tailored and effective prevention. In particular, because adolescents are frequent and avid users of various types of latest technology, computers and smartphones among other technologies could potentially be powerful tools in the primary prevention of substance abuse [218]. Thus, we are quite confident that future research should be focused more on leveraging the use of current CHIT platforms such as mobiles and social media to enhance the outreach of substance abuse prevention programs. 

### Limitations

This study has multiple strengths and is unique in its approach to map the changing trends in the use of CHIT for substance abuse prevention. It covers a long period and spans across the globe. By design, it did not capture technological interventions for alcohol prevention, as a stand-alone review on the prevention of alcohol abuse would be more appropriate. It is also seen that there was a gap in the literature between 1990 and 2008, no studies during this period were included, and this could be attributed to the stringent eligibility criteria for this review. For study limitations, the review solely focuses on primary prevention and hence, fails to capture the use of CHIT in secondary prevention and its role in treatment of substance use.

The focus of the study on primary prevention of substance abuse necessitated the exclusion of a large number of studies; however, there is an opportunity to follow on with subsequent studies to fill this gap. For example, by design, the study did not capture the effects and correlation between substance use and comorbidities such as HIV and risky health behaviors and the use of CHIT to either treat or prevent either of the repercussions of these correlations. It did not include studies conducted in special populations such as cancer patients and patients with HIV, AIDS, or other STDs. This review also did not include a large number of studies that explored the use of mobile and social media platforms as vehicles of intervention delivery, as opposed to providing prevention programs. This again could be attributed to the stringent eligibility criteria.

### Conclusions

This review shows a gradual shift in the types and use of CHIT platforms over the past few decades for substance abuse prevention. It captures the progression from mass media–based interventions toward Web-based intervention and the current trends that head toward a greater emphasis on telehealth- and mHealth-based interventions while emphasizing the need for further development and study of these interventions. It also highlights the gradual shift in consumer and participant behavior, wherein preferences have slowly moved from face-to-face interactions toward more Web- and technology-based platforms, given the anonymity and the vast outreach that these platforms offer. Studies included in this review found these technologies to be effective and cost-effective in real-world settings and contexts. Taking into account the familiarity and ease of use of these CHIT platforms among adults and youth alike, we now have an opportunity to further leverage these platforms for substance use prevention.

## Appendix

Multimedia Appendix 1 Search terms used.

Multimedia Appendix 2 Summary of articles by study design, CHIT used, and outcomes.

Multimedia Appendix 3 Details on articles included in the reviews cited in the manuscript.

## Abbreviations

CHIT: consumer health information technology
mHealth: mobile health
NPS: new psychoactive substances
RCT: randomized controlled trials
STDs: sexually transmitted diseases
WHO: World Health Organization

## References

[1] Columbia CASA (2012). Center On Addiction.

[2] (2017). National Institute on Drug Abuse.

[3] (2018). American Addiction Centers.

[4] Rehm J, Mathers C, Popova S, Thavorncharoensap M, Teerawattananon Y, Patra J (2009). Global burden of disease and injury and economic cost attributable to alcohol use and alcohol-use disorders. Lancet.

[5] Centers for Disease Control and Prevention (2014). Centers for Disease Control and Prevention.

[6] National Drug Intelligence Center (2011). United States Department of Jusitce.

[7] Gianluca QG (2017). European Parliament.

[8] Abuse N (2017). National Institute of Drug Abuse.

[9] Sloboda Z, Bukoski WJ (2006). The Emerging Science of Drug Abuse Prevention. Handbook of drug abuse prevention.

[10] National Institute on Alcohol Abuse and Alcoholism (2005). National Institute of Health.

[11] Medina-Mora M (2005). Prevention of substance abuse: a brief overview. World Psychiatry.

[12] (2018). Substance Abuse and Mental Health Services Administration.

[13] Gibbons M, Wilson R, Samal L, Lehmann C, Dickersin K, Lehmann H, Aboumatar H, Finkelstein J, Shelton E, Sharma R, Bass EB (2011). Consumer health informatics: results of a systematic evidence review and evidence based recommendations. Transl Behav Med.

[14] Or CK, Karsh B, Severtson DJ, Burke LJ, Brown RL, Brennan PF (2011). Factors affecting home care patients' acceptance of a web-based interactive self-management technology. J Am Med Inform Assoc.

[15] Gustafson DH, Hawkins RP, Boberg EW, McTavish F, Owens B, Wise M, Berhe H, Pingree S (2002). CHESS: 10 years of research and development in consumer health informatics for broad populations, including the underserved. Int J Med Inform.

[16] Lewis D, Eysenbach G, Kukafka R (2005). Consumer Health Informatics: Informing Consumers and Improving Health Care.

[17] Tao D, Wang T, Wang T, Liu S, Qu X (2017). Effects of consumer-oriented health information technologies in diabetes management over time: a systematic review and meta-analysis of randomized controlled trials. J Am Med Inform Assoc.

[18] Or C, Karsh B (2009). A systematic review of patient acceptance of consumer health information technology. J Am Med Inform Assoc.

[19] Finkelstein J, Knight A, Marinopoulos S, Gibbons MC, Berger Z, Aboumatar H, Wilson RF, Lau BD, Sharma R, Bass EB (2012). Enabling patient-centered care through health information technology. Evid Rep Technol Assess (Full Rep).

[20] (2018). PEW Research Center.

[21] PEW Research Center.

[22] World Health Organisation.

[23] Marsch LA, Borodovsky JT (2016). Technology-based interventions for preventing and treating substance use among youth. Child Adolesc Psychiatr Clin N Am.

[24] Moore BA, Fazzino T, Garnet B, Cutter CJ, Barry DT (2011). Computer-based interventions for drug use disorders: a systematic review. J Subst Abuse Treat.

[25] Young SD, Swendeman D, Holloway IW, Reback CJ, Kao U (2015). Use of technology to address substance use in the context of HIV: a systematic review. Curr HIV/AIDS Rep.

[26] Feingold PC, Knapp ML (1977). Anti-drug abuse commercials. J Commun.

[27] Miller MC, Cantor AB, Larisey L, Murphy E (1981). Comparison of media for substance abuse education in rural communities. Int J Addict.

[28] Milne HB, Butt TW (1975). The critical assessment of the T.V. film “The Drug Takers”. Br J Addict Alcohol Other Drugs.

[29] Pickens KA (1984). The use of films in drug education--a review. Health Educ J.

[30] Sussman S, Flay B, Sobel J, Rauch J, Hansen W, Johnson C (1987). Viewing and evaluation of a televised drug education program by students previously or concurrently exposed to school-based substance abuse prevention programming. Health Educ Res.

[31] Arksey H, O'Malley L (2005). Scoping studies: towards a methodological framework. Int J Soc Res Methodol.

[32] Levac D, Colquhoun H, O'Brien KK (2010). Scoping studies: advancing the methodology. Implement Sci.

[33] Faulkner GE, Grootendorst P, Nguyen VH, Andreyeva T, Arbour-Nicitopoulos K, Auld MC, Cash SB, Cawley J, Donnelly P, Drewnowski A, Dubé L, Ferrence R, Janssen I, Lafrance J, Lakdawalla D, Mendelsen R, Powell LM, Traill WB, Windmeijer F (2011). Economic instruments for obesity prevention: results of a scoping review and modified Delphi survey. Int J Behav Nutr Phys Act.

[34] Bottorff JL, Haines-Saah R, Kelly MT, Oliffe JL, Torchalla I, Poole N, Greaves L, Robinson CA, Ensom MH, Okoli CT, Phillips JC (2014). Gender, smoking and tobacco reduction and cessation: a scoping review. Int J Equity Health.

[35] Kanter D (1971). The drug scene: current research. Public Opin Q.

[36] O'Keefe M (1971). The Anti-Smoking Commercials: A Study of Television's Impact on Behavio. Public Opin Q.

[37] Fejer D, Smart R, Whitehead P, Laforest L (1971). Sources of information about drugs among high school students. Public Opin Q.

[38] Hanneman G (1972). Sources of Drug Abuse Information on the College Campus.

[39] Linsky AS (1970). The changing public views of alcoholism. Q J Stud Alcohol.

[40] Lipp M, Benson S, Taintor Z (1971). Marijuana use by medical students. Am J Psychiatry.

[41] Pollock SH (2012). Attitudes of medical students toward marijuana. J Psychoactive Drugs.

[42] Grant J (1971). Drug education based on a knowledge, attitude, and experience study. J Sch Health.

[43] Zajonc RB (1968). Attitudinal effects of mere exposure. J Pers Soc Psychol.

[44] Amendolara F (1995). Modifying attitudes towards drugs in seventh grade students. J Drug Educ.

[45] Braxton ER, Yonker RJ (1973). Does being urban, poor, black, or female affect youth's knowledge and-or attitudes relating to drugs?. J Sch Health.

[46] Einstein S, Lavehar M, Garitano WW (1972). Drug abuse education and the multiplier effect: an experience in training 109 teachers. J Sch Health.

[47] Einstein S, Lavenhar MA, Wolfson EA, Louria DB, Quinones MA, McAteer G (1995). The training of teachers for drug abuse education programs: preliminary considerations. J Drug Educ.

[48] Richardson DW, Nader PR, Roghmann KJ, Friedman S (1972). Attitudes of fifth grade students to illicit psychoactive drugs. J Sch Health.

[49] Irwin R, Creswell W, Stauffer D (1970). The effect of the teacher and three different Classroom approaches on seventh grade students? Knowledge, attitudes and beliefs about smoking*. J Sch Health.

[50] Levitt L, Baganz P, Blachly P (1963). A study of employee attitudes toward patients in a hospital for the treatment of drug addiction. Psychiatr Q.

[51] Shaw CT (1972). Knowledge and attitude responses of college students toward controversial social health issues. J Sch Health.

[52] Barcus F (1973). Drug advertising on television. Drug Use in America: Problem in Perspective.

[53] Hanneman G, Eisenstock B, Weinbeck W (1977). The medicine man message: an evaluation of a California state office of narcotics and drug abuse prevention campaign to inform the public of the dangers of prescription and over-the -counter drug misuse.

[54] Warner KE (1977). The effects of the anti-smoking campaign on cigarette consumption. Am J Public Health.

[55] Wotring C, Heald G, Carpenter C (1977). Evaluation of the Florida drug abuse campaign (1976-1977).

[56] Wallack L (1978). An assessment of drinking patterns, problems., knowledge and attidtudes in three Northern California communities. Social research group.

[57] Brecher EM (1973). Licit and Illicit Drugs: The Consumers Union Report on Narcotics, Stimulants, Depressants, Inhalants, Hallucinogens, and Marijuana - Including Caffeine, Nicotine, and Alcohol.

[58] Lazarsfeld P, Merton RK, Schramm W, Roberts D (1971). Mass communication, popular taste,organized social aciton. The process and effects of mass communication. Revised edition.

[59] Fishbein F (1977). Consumer beliefs and behavior with respect to cigarette smoking : a critical analysis of the public literature. A report prepared for the staff of the Federal Trade Commission.

[60] Department of national health and welfare (1977). Long range health planning b, non-medical use of d. Smoking and health in Canada.

[61] Hanneman G, McEwen W (2016). Televised drug abuse appeals: a content analysis. Journal Mass Commun Q.

[62] Schlegel R (1995). The role of persuasive communications in drug dissuasion. J Drug Educ.

[63] Dembo R, Schmeidler J, Lipton DS, Babst DV, Diamond SC, Spielman CR, Bergman PJ, Koval M, Miran MD, Stephens RC (1979). A survey of students' awareness of and attitudes toward drug abuse prevention programs in New York State, winter 1974/75. Int J Addict.

[64] Kline JA (1995). Evaluation of a Multimedia Drug Education Program. J Drug Educ.

[65] Dembo R, Miran M, Babst DV, Schmeidler J (1977). The believability of the media as sources of information on drugs. Int J Addict.

[66] Smart R, Fejer D (1972). Credibility of sources of drug information for high school students. J Drug Issues.

[67] Milavsky J, Pekowsky B, Stipp H (1975). TV drug advertising and proprietary and illicit drug use among teenage boys*. Public Opin Q.

[68] Payne D (1976). The Relationship between Television Advertising and Drug Abuse among Youth: Fancy and Fact.

[69] Kohn P, Snook S (1995). Balanced vs. one-sided communications about drugs. J Drug Educ.

[70] Wotring Ce, Heald G, Carpenter Ct, Schmeling D (1995). Attacking the drug norm: effects of the 1976–77 Florida Drug Abuse TV Campaign. J Drug Educ.

[71] Schmeling D, Wotring C (2016). Agenda-setting effects of drug abuse public service ads. J Q.

[72] Hanneman G (1978). The medicine man message: a delayed effects evaluation of a California state office of narcotics and drug abuse prevention campaign to inform the public of the dangers of prescription and over-the -counter drug misuse. University of Southern California, Center for communications policy research.

[73] Sandmaier M (1980). Sandmaier M. Myths and messages: using the media as a prevention tool, in the women next door: summary proceedings of a symposium on the subject of drugs and the modern women. US journal of drug and alcohol dependence.

[74] Capalaces R, Starr J (1973). The negative message of anti-drug spots: does it get across?. Public Telecommunications Review.

[75] Delaney R (1981). Florida study looks at effects of media messages. National Institute of Alcohol Abuse and Alcoholism information and feature service.

[76] Dickman FB, Keil TJ (1977). Public television and public health. The case of alcoholism. J Stud Alcohol.

[77] Field T, Deitrick S, Hersey J, Probst J, Theologus G (1983). Implementing public education campaigns:lessons from alcohol abuse prevention. Summary report to NIAAA.

[78] Hanneman GJ, McEwen WJ, Coyne SA (1973). Public service advertising on television. Journal of Broadcasting.

[79] Morrison A, Kline F, Miller P, Ostman R (1976). Aspects of adolescent information acquisition about drugs alcohol topics. Communication Research and Drug Education.

[80] Plant MA, Pirie F, Kreitman N (1979). Evaluation of the Scottish Health Education Unit's 1976 campaign on alcoholism. Soc Psychiatry.

[81] Rappeport M, Labow P (1975). The public evaluates the NIAAA public education campaign: a sudy for the U.S. department of health, education, welfare, public health service, alcohol, drug abuse, and mental health administration.

[82] Trager R, Ostman R (1976). Adolescent reactions to educational media messages regarding drug education. Communication research and drug education.

[83] Wong M, Barbatsis G (1976). Attitude and information change effected by drug education via broadcast television and group viewing. J Drug Education.

[84] Weimer J (1976). The effects of film treatments on attitudes that correlated with drug-behavior.

[85] English GE (1972). The effectiveness of emotional appeal versus fact-giving drug educational films. J Sch Health.

[86] Swift B, Dorn N, Thompson A (1974). Evaluation of drug education : findings of a national research study of effects on secondary school students of five types of lessons given by teachers.

[87] Thornell J (1976). The construction and evalution of drug education of a drug education programme for third grade students.

[88] Sohn M (1976). Change in factual knowledge and reported use of illicit drugs resulting from the viewing of a motion picture.

[89] Taussig W (1978). The effects of a family life program and a drug education program on the self esteem of fifth grade children.

[90] Rosengren K, Wenner L, Palmgreen P (1985). Media Gratifications Research: Current Perspectives.

[91] Flay B, Pentz M, Johnson C, Sussman S, Leather G (1986). Reaching children with mass media health promotion programs: the relative effectivenss of an advertising campaign, a community-based programa school-based program. Health evaluation and the media.

[92] Flay B (1985). Psychosocial approaches to smoking prevention: a review of findings. Health Psychol.

[93] Flay BR (1987). Mass media and smoking cessation: a critical review. Am J Public Health.

[94] National Coalition of Hispanic Health and Human Services Organization (1988). Early intervention with Hispanic youth.

[95] Amaro H, Campa R, Coffman G, Heeren T (1989). Initiation of substance abuse among Mexican American, Cuban American, and Puerto Rican adolescents and young adults:findings from the Hispanic HANES. Am J Public Health.

[96] Booth M, Castro F, Anglin M, Glick R, Moore J (1989). What do we know about Hispanic substance abuse? a review of the literature. Drugs in Hispanic communities.

[97] Caetano R, Martinez R (1987). Alcohol use in Madrid and among U.S. Hispanics.

[98] Buller DB, Borland R, Woodall WG, Hall JR, Hines JM, Burris-Woodall P, Cutter GR, Miller C, Balmford J, Starling R, Ax B, Saba L (2008). Randomized trials on consider this, a tailored, internet-delivered smoking prevention program for adolescents. Health Educ Behav.

[99] Norman CD, Maley O, Li X, Skinner HA (2008). Using the internet to assist smoking prevention and cessation in schools: a randomized, controlled trial. Health Psychol.

[100] Prokhorov AV, Kelder SH, Shegog R, Murray N, Peters Jr R, Agurcia-Parker C, Cinciripini PM, de Moor C, Conroy JL, Hudmon KS, Ford KH, Marani S (2008). Impact of A Smoking Prevention Interactive Experience (ASPIRE), an interactive, multimedia smoking prevention and cessation curriculum for culturally diverse high-school students. Nicotine Tob Res.

[101] Vogl L, Teesson M, Andrews G, Bird K, Steadman B, Dillon P (2009). A computerized harm minimization prevention program for alcohol misuse and related harms: randomized controlled trial. Addiction.

[102] Newton NC, Vogl LE, Teesson M, Andrews G (2009). CLIMATE Schools: alcohol module: cross-validation of a school-based prevention programme for alcohol misuse. Aust N Z J Psychiatry.

[103] Newton NC, Andrews G, Teesson M, Vogl LE (2009). Delivering prevention for alcohol and cannabis using the Internet: a cluster randomised controlled trial. Prev Med.

[104] Newton NC, Teesson M, Vogl LE, Andrews G (2010). Internet-based prevention for alcohol and cannabis use: final results of the Climate Schools course. Addiction.

[105] Koning IM, Vollebergh WA, Smit F, Verdurmen JEE, Van Den Eijnden RJ, Ter Bogt TF, Stattin H, Engels RC (2009). Preventing heavy alcohol use in adolescents (PAS): cluster randomized trial of a parent and student intervention offered separately and simultaneously. Addiction.

[106] Aveyard P, Sherratt E, Almond J, Lawrence T, Lancashire R, Griffin C, Cheng KK (2001). The change-in-stage and updated smoking status results from a cluster-randomized trial of smoking prevention and cessation using the transtheoretical model among British adolescents. Prev Med.

[107] Marsch LA, Bickel WK, Grabinski MJ (2007). Application of interactive, computer technology to adolescent substance abuse prevention and treatment. Adolesc Med State Art Rev.

[108] Duncan TE, Duncan SC, Beauchamp N, Wells J, Ary DV (2000). Development and evaluation of an interactive CD-ROM refusal skills program to prevent youth substance use: “refuse to use”. J Behav Med.

[109] Lord S, D’Amante D (2007). Efficacy of online alcohol and other drug prevention for early adolescents. J Adolesc Health.

[110] Budney AJ, Fearer S, Walker DD, Stanger C, Thostenson J, Grabinski M, Bickel WK (2011). An initial trial of a computerized behavioral intervention for cannabis use disorder. Drug Alcohol Depend.

[111] Gilbert P, Ciccarone D, Gansky SA, Bangsberg DR, Clanon K, McPhee SJ, Calderón SH, Bogetz A, Gerbert B (2008). Interactive “Video Doctor” counseling reduces drug and sexual risk behaviors among HIV-positive patients in diverse outpatient settings. PLoS One.

[112] Kay-Lambkin FJ, Baker AL, Lewin TJ, Carr VJ (2009). Computer-based psychological treatment for comorbid depression and problematic alcohol and/or cannabis use: a randomized controlled trial of clinical efficacy. Addiction.

[113] Lee CM, Neighbors C, Kilmer JR, Larimer ME (2010). A brief, web-based personalized feedback selective intervention for college student marijuana use: a randomized clinical trial. Psychol Addict Behav.

[114] Tossmann H, Jonas B, Tensil M, Lang P, Strüber E (2011). A controlled trial of an internet-based intervention program for cannabis users. Cyberpsychol Behav Soc Netw.

[115] Williams C, Griffin KW, Macaulay AP, West TL, Gronewold E (2005). Efficacy of a drug prevention CD-ROM intervention for adolescents. Subst Use Misuse.

[116] Bersamin M, Paschall MJ, Fearnow-Kenney M, Wyrick D (2007). Effectiveness of a web-based alcohol-misuse and harm-prevention course among high- and low-risk students. J Am Coll Health.

[117] Bingham CR, Barretto AI, Walton MA, Bryant CM, Shope JT, Raghunathan TE (2011). Efficacy of a web-based, tailored, alcohol prevention/intervention program for college students: 3-month follow-up. J Drug Educ.

[118] Bishop D, Bryant KS, Giles SM, Hansen WB, Dusenbury L (2006). Simplifying the delivery of a prevention program with web-based enhancements. J Prim Prev.

[119] Croom K, Lewis D, Marchell T, Lesser ML, Reyna VF, Kubicki-Bedford L, Feffer M, Staiano-Coico L (2009). Impact of an online alcohol education course on behavior and harm for incoming first-year college students: short-term evaluation of a randomized trial. J Am Coll Health.

[120] Di Noia J, Schwinn TM, Dastur ZA, Schinke SP (2003). The relative efficacy of pamphlets, CD-ROM, and the Internet for disseminating adolescent drug abuse prevention programs: an exploratory study. Prev Med.

[121] Epstein J, Collins K, Thomson N, Pancella T, Pauley D (2007). The doubles: evaluation of a substance abuse education curriculum for elementary school students. J Child Adolesc Subst Abuse.

[122] Hecht ML, Marsiglia FF, Elek E, Wagstaff DA, Kulis S, Dustman P, Miller-Day M (2003). Culturally grounded substance use prevention: an evaluation of the keepin' it R.E.A.L. curriculum. Prev Sci.

[123] Hustad JT, Barnett NP, Borsari B, Jackson KM (2010). Web-based alcohol prevention for incoming college students: a randomized controlled trial. Addict Behav.

[124] Koning IM, van den Eijnden RJ, Verdurmen JE, Engels RC, Vollebergh WA (2011). Long-term effects of a parent and student intervention on alcohol use in adolescents: a cluster randomized controlled trial. Am J Prev Med.

[125] Kypri K, Saunders JB, Williams SM, McGee RO, Langley JD, Cashell-Smith ML, Gallagher SJ (2004). Web-based screening and brief intervention for hazardous drinking: a double-blind randomized controlled trial. Addiction.

[126] Moore MJ, Soderquist J, Werch C (2005). Feasibility and efficacy of a binge drinking prevention intervention for college students delivered via the Internet versus postal mail. J Am Coll Health.

[127] Neighbors C, Lewis MA, Atkins DC, Jensen MM, Walter T, Fossos N, Lee CM, Larimer ME (2010). Efficacy of web-based personalized normative feedback: a two-year randomized controlled trial. J Consult Clin Psychol.

[128] Rohrbach LA, Gunning M, Sun P, Sussman S (2010). The Project Towards No Drug Abuse (TND) dissemination trial: implementation fidelity and immediate outcomes. Prev Sci.

[129] Schinke SP, Cole KC, Fang L (2009). Gender-specific intervention to reduce underage drinking among early adolescent girls: a test of a computer-mediated, mother-daughter program. J Stud Alcohol Drugs.

[130] Schinke SP, Schwinn TM, Di Noia J, Cole KC (2004). Reducing the risks of alcohol use among urban youth: three-year effects of a computer-based intervention with and without parent involvement. J Stud Alcohol.

[131] Walters ST, Vader AM, Harris TR (2007). A controlled trial of web-based feedback for heavy drinking college students. Prev Sci.

[132] Warren J, Hecht M, Wagstaff D, Elek E, Ndiaye K, Dustman P, Marsiglia Ff (2006). Communicating prevention: the effects of the keepin' it REAL classroom videotapes and televised PSAs on middle-school students' substance use. J Appl Commun Res.

[133] de Josselin de Jong JS, Candel M, Segaar D, Cremers H, de Vries H (2014). Efficacy of a web-based computer-tailored smoking prevention intervention for Dutch adolescents: randomized controlled trial. J Med Internet Res.

[134] Doumas DM, Esp S, Turrisi R, Hausheer R, Cuffee C (2014). A test of the efficacy of a brief, web-based personalized feedback intervention to reduce drinking among 9th grade students. Addict Behav.

[135] Doumas DM, Hausheer R, Esp S, Cuffee C (2014). Reducing alcohol use among 9th grade students: 6 month outcomes of a brief, Web-based intervention. J Subst Abuse Treat.

[136] Vogl LE, Newton NC, Champion KE, Teesson M (2014). A universal harm-minimisation approach to preventing psychostimulant and cannabis use in adolescents: a cluster randomised controlled trial. Subst Abuse Treat Prev Policy.

[137] Malmberg M, Kleinjan M, Overbeek G, Vermulst A, Lammers J, Monshouwer K, Vollebergh WA, Engels RC (2015). Substance use outcomes in the Healthy School and Drugs program: results from a latent growth curve approach. Addict Behav.

[138] Malmberg M, Kleinjan M, Overbeek G, Vermulst A, Monshouwer K, Lammers J, Vollebergh WA, Engels RC (2014). Effectiveness of the 'Healthy School and Drugs' prevention programme on adolescents' substance use: a randomized clustered trial. Addiction.

[139] Velicer WF, Redding CA, Paiva AL, Mauriello LM, Blissmer B, Oatley K, Meier KS, Babbin SF, McGee H, Prochaska JO, Burditt C, Fernandez AC (2013). Multiple behavior interventions to prevent substance abuse and increase energy balance behaviors in middle school students. Transl Behav Med.

[140] Bannink R, Broeren S, Joosten-van Zwanenburg Evelien, van As E, van de Looij-Jansen P, Raat H (2014). Effectiveness of a Web-based tailored intervention (E-health4Uth) and consultation to promote adolescents' health: randomized controlled trial. J Med Internet Res.

[141] Rundle-Thiele S, Schuster L, Dietrich T, Russell-Bennett R, Drennan J, Leo C, Connor Jp (2015). Maintaining or changing a drinking behavior? GOKA's short-term outcomes. J Bus Res.

[142] Walton MA, Resko S, Barry KL, Chermack ST, Zucker RA, Zimmerman MA, Booth BM, Blow FC (2014). A randomized controlled trial testing the efficacy of a brief cannabis universal prevention program among adolescents in primary care. Addiction.

[143] Bauman KE, LaPrelle J, Brown JD, Koch GG, Padgett CA (1991). The influence of three mass media campaigns on variables related to adolescent cigarette smoking: results of a field experiment. Am J Public Health.

[144] Bauman KE, Brown JD, Bryan ES, Fisher LA, Padgett CA, Sweeney JM (1988). Three mass media campaigns to prevent adolescent cigarette smoking. Prev Med.

[145] Bauman K, Padgett C, Koch G (1989). A media-based campaign to encourage personal communication among adolescents about not smoking cigarettes: participation, selection and consequences. Health Educ Res.

[146] Brown JD, Bauman KE, Padgett CA (1990). A validity problem in measuring exposure to mass media campaigns. Health Educ Q.

[147] L J, Bauman KE, Koch GG (2016). High intercommunity variation in adolescent cigarette smoking in a 10-community field experiment. Eval Rev.

[148] Fallin A, Neilands TB, Jordan JW, Hong JS, Ling PM (2015). Wreaking “havoc” on smoking: social branding to reach young adult “partiers” in Oklahoma. Am J Prev Med.

[149] Flay BR, Miller TQ, Hedeker D, Siddiqui O, Britton CF, Brannon BR, Johnson CA, Hansen WB, Sussman S, Dent C (1995). The television, school, and family smoking prevention and cessation project. VIII. Student outcomes and mediating variables. Prev Med.

[150] Flay BR, Brannon BR, Johnson CA, Hansen WB, Ulene AL, Whitney-Saltiel DA, Gleason LR, Sussman S, Gavin MD, Glowacz KM (1988). The television school and family smoking prevention and cessation project. 1. Theoretical basis and program development. Prev Med.

[151] Sussman S, Brannon BR, Flay BR, Gleason L, Senor S, Sobol DF, Hansen WB, Johnson CA (1986). The television, school and family smoking prevention/cessation project. II. Formative evaluation of television segments by teenagers and parents – implications for parental involvement in drug education. Health Educ Res.

[152] Sussman S, Dent CW, Brannon BR, Glowacz K, Gleason LR, Ullery S, Hansen WB, Johnson CA, Flay BR (1989). The television, school and family smoking prevention/cessation project. IV. Controlling for program success expectancies across experimental and control conditions. Addict Behav.

[153] Flynn BS, Worden JK, Secker-Walker RH, Badger GJ, Geller BM (2013). Cigarette smoking prevention effects of mass media and school interventions targeted to gender and age groups. Journal of Health Education.

[154] Flynn BS, Worden JK, Secker-Walker RH, Badger GJ, Geller BM, Costanza MC (1992). Prevention of cigarette smoking through mass media intervention and school programs. Am J Public Health.

[155] Flynn BS, Worden JK, Secker-Walker RH, Pirie PL, Badger GJ, Carpenter JH (1997). Long-term responses of higher and lower risk youths to smoking prevention interventions. Prev Med.

[156] Flynn BS, Worden JK, Secker-Walker RH, Pirie PL, Badger GJ, Carpenter JH, Geller BM (1994). Mass media and school interventions for cigarette smoking prevention: effects 2 years after completion. Am J Public Health.

[157] Secker-Walker RH, Worden JK, Holland RR, Flynn BS, Detsky AS (1997). A mass media programme to prevent smoking among adolescents: costs and cost effectiveness. Tob Control.

[158] Worden JK, Flynn BS (2000). Effective use of mass media to prevent cigarette smoking. J Public Health Manag Pract.

[159] Worden JK, Flynn BS, Geller BM, Chen M, Shelton LG, Secker-Walker RH, Solomon DS, Solomon LJ, Couchey S, Costanza MC (1988). Development of a smoking prevention mass media program using diagnostic and formative research. Prev Med.

[160] Worden JK, Flynn BS, Solomon LJ, Secker-Walker RH, Badger GJ, Carpenter JH (1996). Using mass media to prevent cigarette smoking among adolescent girls. Health Educ Q.

[161] Flynn BS, Worden JK, Bunn JY, Solomon LJ, Ashikaga T, Connolly SW, Ramirez AG (2010). Mass media interventions to reduce youth smoking prevalence. Am J Prev Med.

[162] Solomon LJ, Bunn JY, Flynn BS, Pirie PL, Worden JK, Ashikaga T (2009). Mass media for smoking cessation in adolescents. Health Educ Behav.

[163] Hafstad A, Aaro L (1997). Activating interpersonal influence through provocative appeals: evaluation of a mass media-based antismoking campaign targeting adolescents. Health Commun.

[164] Hafstad A (1997). Provocative anti-smoking appeals in mass-media campaigns: an intervention study on adolescent smoking.

[165] Hafstad A, Aarø LE, Engeland A, Andersen A, Langmark F, Stray-Pedersen B (1997). Provocative appeals in anti-smoking mass media campaigns targeting adolescents--the accumulated effect of multiple exposures. Health Educ Res.

[166] Hafstad A (1997). Use of provocative emotional appeals in a mass media campaign designed to prevent smoking among adolescents. Eur J Public Health.

[167] Longshore D, Ghosh-Dastidar B, Ellickson PL (2006). National Youth Anti-Drug Media Campaign and school-based drug prevention: evidence for a synergistic effect in ALERT Plus. Addict Behav.

[168] Ellickson PL, McCaffrey DF, Ghosh-Dastidar B, Longshore DL (2003). New inroads in preventing adolescent drug use: results from a large-scale trial of project ALERT in middle schools. Am J Public Health.

[169] Ghosh-Dastidar B, Longshore DL, Ellickson PL, McCaffrey DF (2004). Modifying pro-drug risk factors in adolescents: results from project ALERT. Health Educ Behav.

[170] Longshore D, Ellickson PL, McCaffrey DF, St Clair P (2007). School-based drug prevention among at-risk adolescents: effects of ALERT plus. Health Educ Behav.

[171] Worden J, Flynn B (1983). Using television messages to prevent smoking among adolescents.

[172] Stotts AL, Diclemente CC, Dolan-Mullen P (2002). One-to-one: a motivational intervention for resistant pregnant smokers. Addict Behav.

[173] Rigotti NA, Park ER, Regan S, Chang Y, Perry K, Loudin B, Quinn V (2006). Efficacy of telephone counseling for pregnant smokers: a randomized controlled trial. Obstet Gynecol.

[174] Peterson AV, Kealey KA, Mann SL, Marek PM, Ludman EJ, Liu J, Bricker JB (2009). Group-randomized trial of a proactive, personalized telephone counseling intervention for adolescent smoking cessation. J Natl Cancer Inst.

[175] Severson HH, Peterson AL, Andrews JA, Gordon JS, Cigrang JA, Danaher BG, Hunter CM, Barckley M (2009). Smokeless tobacco cessation in military personnel: a randomized controlled trial. Nicotine Tob Res.

[176] Bastian LA, Fish LJ, Peterson BL, Biddle AK, Garst J, Lyna P, Molner S, Bepler G, Kelley M, Keefe FJ, McBride CM (2013). Assessment of the impact of adjunctive proactive telephone counseling to promote smoking cessation among lung cancer patients' social networks. Am J Health Promot.

[177] Jiménez-Muro A, Nerín I, Samper P, Marqueta A, Beamonte A, Gargallo P, Oros D, Rodríguez G (2013). A proactive smoking cessation intervention in postpartum women. Midwifery.

[178] Woodruff SI, Conway TL, Edwards CC, Elliott SP, Crittenden J (2007). Evaluation of an Internet virtual world chat room for adolescent smoking cessation. Addict Behav.

[179] Becker J, Haug S, Sullivan R, Schaub MP (2014). Effectiveness of different Web-based interventions to prepare co-smokers of cigarettes and cannabis for double cessation: a three-arm randomized controlled trial. J Med Internet Res.

[180] Mason MJ, Campbell L, Way T, Keyser-Marcus L, Benotsch E, Mennis J, Zhang J, King L, May J, Stembridge DR (2015). Development and outcomes of a text messaging tobacco cessation intervention with urban adolescents. Subst Abus.

[181] Blankers M, Koeter MW, Schippers GM (2011). Internet therapy versus internet self-help versus no treatment for problematic alcohol use: a randomized controlled trial. J Consult Clin Psychol.

[182] Brown RL, Saunders LA, Bobula JA, Mundt MP, Koch PE (2007). Randomized-controlled trial of a telephone and mail intervention for alcohol use disorders: three-month drinking outcomes. Alcohol Clin Exp Res.

[183] Borsari B, Short EE, Mastroleo NR, Hustad JTP, Tevyaw TO, Barnett NP, Kahler CW, Monti PM (2014). Phone-delivered brief motivational interventions for mandated college students delivered during the summer months. J Subst Abuse Treat.

[184] LaChance H, Feldstein ES, Bryan AD, Hutchison KE (2009). What makes group MET work? A randomized controlled trial of college student drinkers in mandated alcohol diversion. Psychol Addict Behav.

[185] Wongpakaran T, Petcharaj K, Wongpakaran N, Sombatmai S, Boripuntakul T, Intarakamhaeng D, Wannarit K (2011). The effect of telephone-based intervention (TBI) in alcohol abusers: a pilot study. J Med Assoc Thai.

[186] D'Amico EJ, Hunter SB, Miles JNV, Ewing BA, Osilla KC (2013). A randomized controlled trial of a group motivational interviewing intervention for adolescents with a first time alcohol or drug offense. J Subst Abuse Treat.

[187] D'Amico EJ, Houck JM, Hunter SB, Miles JNV, Osilla KC, Ewing BA (2015). Group motivational interviewing for adolescents: change talk and alcohol and marijuana outcomes. J Consult Clin Psychol.

[188] Nyamathi A, Shoptaw S, Cohen A, Greengold B, Nyamathi K, Marfisee M, de CV, Khalilifard F, George D, Leake B (2010). Effect of motivational interviewing on reduction of alcohol use. Drug Alcohol Depend.

[189] Nyamathi AM, Nandy K, Greengold B, Marfisee M, Khalilifard F, Cohen A, Leake B (2011). Effectiveness of intervention on improvement of drug use among methadone maintained adults. J Addict Dis.

[190] Suffoletto B, Kristan J, Callaway C, Kim KH, Chung T, Monti PM, Clark DB (2014). A text message alcohol intervention for young adult emergency department patients: a randomized clinical trial. Ann Emerg Med.

[191] Suffoletto B, Kristan J, Chung T, Jeong K, Fabio A, Monti P, Clark DB (2015). An interactive text message intervention to reduce binge drinking in young adults: a randomized controlled trial with 9-Month Outcomes. PLoS One.

[192] Gates PJ, Norberg MM, Copeland J, Digiusto E (2012). Randomized controlled trial of a novel cannabis use intervention delivered by telephone. Addiction.

[193] Madigan K, Brennan D, Lawlor E, Turner N, Kinsella A, O'Connor JJ, Russell V, Waddington JL, O'Callaghan E (2013). A multi-center, randomized controlled trial of a group psychological intervention for psychosis with comorbid cannabis dependence over the early course of illness. Schizophr Res.

[194] Stevens J, Hayes J, Pakalnis A (2014). A randomized trial of telephone-based motivational interviewing for adolescent chronic headache with medication overuse. Cephalalgia.

[195] Agyapong VIO, Ahern S, McLoughlin DM, Farren CK (2012). Supportive text messaging for depression and comorbid alcohol use disorder: single-blind randomised trial. J Affect Disord.

[196] Weitzel JA, Bernhardt JM, Usdan S, Mays D, Glanz K (2007). Using wireless handheld computers and tailored text messaging to reduce negative consequences of drinking alcohol. J Stud Alcohol Drugs.

[197] Suffoletto B, Callaway C, Kristan J, Kraemer K, Clark DB (2012). Text-message-based drinking assessments and brief interventions for young adults discharged from the emergency department. Alcohol Clin Exp Res.

[198] Haug S, Schaub MP, Venzin V, Meyer C, John U, Gmel G (2013). A pre-post study on the appropriateness and effectiveness of a Web- and text messaging-based intervention to reduce problem drinking in emerging adults. J Med Internet Res.

[199] Gajecki M, Berman AH, Sinadinovic K, Rosendahl I, Andersson C (2014). Mobile phone brief intervention applications for risky alcohol use among university students: a randomized controlled study. Addict Sci Clin Pract.

[200] Gonzales R, Ang A, Murphy DA, Glik DC, Anglin MD (2014). Substance use recovery outcomes among a cohort of youth participating in a mobile-based texting aftercare pilot program. J Subst Abuse Treat.

[201] Gustafson DH, McTavish FM, Chih M, Atwood AK, Johnson RA, Boyle MG, Levy MS, Driscoll H, Chisholm SM, Dillenburg L, Isham A, Shah D (2014). A smartphone application to support recovery from alcoholism: a randomized clinical trial. JAMA Psychiatry.

[202] Lucht MJ, Hoffman L, Haug S, Meyer C, Pussehl D, Quellmalz A, Klauer T, Grabe HJ, Freyberger HJ, John U, Schomerus G (2014). A surveillance tool using mobile phone short message service to reduce alcohol consumption among alcohol-dependent patients. Alcohol Clin Exp Res.

[203] Shrier LA, Rhoads A, Burke P, Walls C, Blood EA (2014). Real-time, contextual intervention using mobile technology to reduce marijuana use among youth: a pilot study. Addict Behav.

[204] Haug S, Lucht MJ, John U, Meyer C, Schaub MP (2015). A pilot study on the feasibility and acceptability of a text message-based aftercare treatment programme among alcohol outpatients. Alcohol Alcohol.

[205] Gonzalez VM, Dulin PL (2015). Comparison of a smartphone app for alcohol use disorders with an internet-based intervention plus bibliotherapy: A pilot study. J Consult Clin Psychol.

[206] Schwinn TM, Schinke SP, Di Noia J (2010). Preventing drug abuse among adolescent girls: outcome data from an internet-based intervention. Prev Sci.

[207] Wood S, Eckley L, Hughes K, Hardcastle K, Bellis M, Schrooten J, Demetrovics Z, Voorham L (2014). Computer-based programmes for the prevention and management of illicit recreational drug use: a systematic review. Addict Behav.

[208] Hopson L, Wodarski J, Tang N (2015). The effectiveness of electronic approaches to substance abuse prevention for adolescents. J Evid Inf Soc Work.

[209] Champion KE, Newton NC, Barrett EL, Teesson M (2013). A systematic review of school-based alcohol and other drug prevention programs facilitated by computers or the internet. Drug Alcohol Rev.

[210] Newton NC, Andrews G, Champion KE, Teesson M (2014). Universal Internet-based prevention for alcohol and cannabis use reduces truancy, psychological distress and moral disengagement: a cluster randomised controlled trial. Prev Med.

[211] Champion KE, Newton NC, Stapinski L, Slade T, Barrett EL, Teesson M (2016). A cross-validation trial of an Internet-based prevention program for alcohol and cannabis: preliminary results from a cluster randomised controlled trial. Aust N Z J Psychiatry.

[212] Champion KE, Newton NC, Stapinski LA, Teesson M (2016). Effectiveness of a universal internet-based prevention program for ecstasy and new psychoactive substances: a cluster randomized controlled trial. Addiction.

[213] Christoff AO, Boerngen-Lacerda R (2015). Reducing substance involvement in college students: a three-arm parallel-group randomized controlled trial of a computer-based intervention. Addict Behav.

[214] Champion K, Newton N, Teesson M (2016). Prevention of alcohol and other drug use and related harm in the digital age: what does the evidence tell us?. Curr Opin Psychiatry.

[215] Brinn MP, Carson KV, Esterman AJ, Chang AB, Smith BJ (2010). Mass media interventions for preventing smoking in young people. Cochrane Database Syst Rev.

[216] Carson KV, Ameer F, Sayehmiri K, Hnin K, van Agteren JE, Sayehmiri F, Brinn MP, Esterman AJ, Chang AB, Smith BJ (2017). Mass media interventions for preventing smoking in young people. Cochrane Database Syst Rev.

[217] Eiser C, Eiser Jr, Pritchard M (1988). Reactions to Drug Education: a comparison of two videos produced for schools. Br J Addict.

[218] Haug S, Paz Castro R, Meyer C, Filler A, Kowatsch T, Schaub M (2017). A mobile phone-based life skills training program for substance use prevention among adolescents: pre-post study on the acceptance and potential effectiveness of the program, Ready4life. JMIR Mhealth Uhealth.

[219] Deitz D, Cook R, Hendrickson A (2011). Preventing prescription drug misuse: field test of the SmartRx Web program. Subst Use Misuse.

[220] Fang L, Schinke S, Cole K (2010). Preventing substance use among early Asian-American adolescent girls: initial evaluation of a web-based, mother-daughter program. J Adolesc Health.

[221] Terry-McElrath YM, Emery S, Szczypka G, Johnston LD (2011). Potential exposure to anti-drug advertising and drug-related attitudes, beliefs, and behaviors among United States youth, 1995-2006. Addict Behav.

[222] Brannon BR, Dent CW, Flay BR, Smith G, Sussman S, Pentz MA, Johnson CA, Hansen WB (1989). The television, school, and family project. V. The impact of curriculum delivery format on program acceptance. Prev Med.

[223] Schinke S, Fang L, Cole K, Cohen-Cutler S (2011). Preventing substance use among Black and Hispanic adolescent girls: results from a computer-delivered, mother-daughter intervention approach. Subst Use Misuse.

[224] Klisch Y, Bowling KG, Miller LM, Ramos MA (2013). The impact of science education games on prescription drug abuse attitudes among teens: a case study. J Drug Educ.

[225] Schuman SH (1971). Drug perception and the student-teacher gap. Reactions of 428 students and 72 teachers to an experimental trigger film on drugs. J Am Med Assoc.

[226] Flay BR (1986). Mass media linkages with school-based programs for drug abuse prevention. J Sch Health.

[227] Schinke SP, Fang L, Cole KC (2009). Computer-delivered, parent-involvement intervention to prevent substance use among adolescent girls. Prev Med.

[228] Hansen WB, Bishop DC, Bryant KS (2009). Using online components to facilitate program implementation: impact of technological enhancements to all stars on ease and quality of program delivery. Prev Sci.

[229] Andrews J, Gordon J, Hampson S, Gunn B, Christiansen S, Slovic P (2014). Long-term efficacy of click city(r): tobacco: a school-based tobacco prevention program. Nicotine Tob Res.

[230] Kinder BN (2009). Attitudes toward alcohol and drug abuse. II. Experimental data, mass media research, and methodological considerations. International Journal of the Addictions.

[231] Bandy P, President P (1995). Recent literature on drug abuse prevention and mass media: focusing on youth, parents, women and the elderly. J Drug Educ.

[232] Johnson E, Delgado J (1989). Reaching Hispanics with messages to prevent alcohol and other drug abuse. Public Health Rep.

[233] Jiang S, Wu L, Gao X (2017). Beyond face-to-face individual counseling: a systematic review on alternative modes of motivational interviewing in substance abuse treatment and prevention. Addict Behav.

[234] Schinke SP, Fang L, Cole KC (2009). Preventing substance use among adolescent girls: 1-year outcomes of a computerized, mother-daughter program. Addict Behav.

[235] Schwinn T, Schinke S, Hopkins J, Keller B, Liu X (2018). An online drug abuse prevention program for adolescent girls: posttest and 1-year outcomes. J Youth Adolesc.

[236] Moncher MS, Parms CA, Orlandi MA, Schinke SP, Miller SO, Palleja J, Schinke MB (1989). Microcomputer-based approaches for preventing drug and alcohol abuse among adolescents from ethnic-racial minority backgrounds. Comput Human Behav.

[237] Flay BR, Sobel JL (1983). The role of mass media in preventing adolescent substance abuse. NIDA Res Monogr.

[238] Wallack L (2016). Mass media campaigns: the odds against finding behavior change. Health Educ Q.

[239] Wallack L (1980). Mass media and drinking, smoking, and drug taking. Contemporary drug problems.

[240] Barcus F, Jankowski S (2016). Drugs and the mass media. Ann Am Acad Polit Soc Sci.

[241] Andrews J, Gordon J, Hampson S, Christiansen S, Gunn B, Slovic P, Severson HH (2011). Short-term efficacy of Click City®: Tobacco: changing etiological mechanisms related to the onset of tobacco use. Prev Sci.

[242] Kazemi DM, Borsari B, Levine MJ, Li S, Lamberson KA, Matta LA (2017). A systematic review of the mHealth interventions to prevent alcohol and substance abuse. J Health Commun.

[243] (2016). The Evolution of Media. Understanding Media and Culture: An Introduction to Mass Communication.

[244] Marsch L, Bickel W, Badger G (2007). Applying computer technology to substance abuse prevention science: results of a preliminary examination. J Child Adolesc Subst Abuse.

[245] U.S. Department of Health and Human Services (2018). HHS.GOV/OPIOIDS.

