# Supplementary material for: Consumer Health Information Technology in the Prevention of Substance Abuse: Scoping Review
Source: J Med Internet Res. 2019 Jan 30;21(1):e11297. doi: 10.2196/11297 (PMC6372939; doi:10.2196/11297)
Supplement: Multimedia Appendix 1 [file jmir_v21i1e11297_app1.pdf]

Multimedia Appendix: **Systematic Review Search Strategies – technology use in the prevention of substance abuse**

This is a Multimedia Appendix to a full manuscript published in the J Med Internet Res. For full copyright and citation information see http://doi.org/10.2196/preprints.11297

Final search run on 01/26/2018 by Emily F. Gorman
Total references on 01/26/2018: 4393 references
Total following de-duplication: **4135** references

Search Terms used for Pubmed, Embase, Scopus, and Cochrane: 4393 articles found (1809 - present) references retrieved on 01/26/2018

("technology"[Mesh] OR "internet"[Mesh] OR "cell phone"[Mesh] OR "multimedia"[Mesh] OR "computer-assisted instruction"[Mesh] OR "therapy, computer-assisted"[Mesh] OR "mobile applications"[Mesh] OR "computer systems"[Mesh] OR "Telemedicine"[Mesh] OR technology[tiab] OR technologies[tiab] OR social media[tiab] OR facebook[tiab] OR twitter[tiab] OR internet[tiab] OR web-based[tiab] OR mobile phone*[tiab] OR cell phone*[tiab] OR smartphone*[tiab] OR texting[tiab] OR text messag*[tiab] OR mobile app[tiab] OR mobile apps[tiab] OR mobile application*[tiab] OR mhealth[tiab] OR ehealth[tiab] OR telehealth[tiab] OR telemedicine[tiab] OR multimedia[tiab] OR mass media[tiab])

AND

(prevent*[tiab] OR "prevention and control"[subheading] OR "preventive health services"[Mesh])

AND

("substance-related disorders"[Mesh] OR substance abuse[tiab] OR substance misuse[tiab] OR substance addiction[tiab] OR substance use disorder*[tiab] OR drug addiction[tiab] OR drug abuse[tiab] OR drug misuse[tiab] OR drug dependence[tiab] OR drug use disorder[tiab] OR alcohol abuse[tiab] OR alcohol misuse[tiab] OR alcohol dependence[tiab] OR alcohol addiction[tiab] OR alcohol use disorder*[tiab] OR alcoholism[tiab] OR tobacco abuse[tiab] OR tobacco misuse[tiab] OR tobacco dependence[tiab] OR tobacco addiction[tiab] OR tobacco use disorder*[tiab] OR nicotine abuse[tiab] OR nicotine misuse[tiab] OR nicotine dependence[tiab] OR nicotine addiction[tiab] OR nicotine use disorder*[tiab] OR smoking abuse[tiab] OR smoking dependence[tiab] OR smoking addiction[tiab])

('technology':ti,ab OR 'technologies':ti,ab OR 'social media':ti,ab OR 'facebook':ti,ab OR 'twitter':ti,ab OR 'internet':ti,ab OR 'web-based':ti,ab OR 'mobile phone*':ti,ab OR 'cell phone*':ti,ab OR 'texting':ti,ab OR 'text messag*':ti,ab OR 'smartphone*':ti,ab OR 'mobile app':ti,ab OR 'mobile apps':ti,ab OR 'mobile application*':ti,ab OR 'mhealth':ti,ab OR 'ehealth':ti,ab OR 'telehealth':ti,ab OR 'telemedicine':ti,ab OR 'multimedia':ti,ab OR 'mass media':ti,ab OR 'technology'/exp OR 'mass communication'/exp OR 'multimedia'/exp OR 'mobile application'/exp OR 'information technology device'/exp OR 'telehealth'/exp OR 'mhealth'/exp)

AND

('prevention and control'/exp OR 'preventive medicine'/exp OR 'prevent*':ti,ab)

AND

('substance abuse'/exp OR 'drug dependence'/exp OR 'drug abuse'/exp OR 'smoking prevention'/exp OR 'substance abuse':ti,ab OR 'substance misuse':ti,ab OR 'substance addiction':ti,ab OR 'substance dependence':ti,ab OR 'substance use disorder*':ti,ab OR 'drug misuse':ti,ab OR 'drug addiction':ti,ab OR 'drug abuse':ti,ab OR 'drug dependence':ti,ab OR 'drug use disorder*':ti,ab OR 'alcohol abuse':ti,ab OR 'alcohol misuse':ti,ab OR 'alcohol dependence':ti,ab OR 'alcohol use disorder*':ti,ab OR 'alcohol addiction':ti,ab OR 'alcoholism':ti,ab OR 'tobacco abuse':ti,ab OR 'tobacco misuse':ti,ab OR 'tobacco dependence':ti,ab OR 'tobacco addiction':ti,ab OR 'tobacco use disorder*':ti,ab OR 'nicotine abuse':ti,ab OR 'nicotine misuse':ti,ab OR 'nicotine dependence':ti,ab OR 'nicotine addiction':ti,ab OR 'nicotine use disorder*':ti,ab OR 'smoking abuse':ti,ab OR 'smoking dependence':ti,ab OR 'smoking addiction':ti,ab)

AND [embase]/lim NOT ([embase]/lim AND [medline]/lim)

technology OR technologies OR "social media" OR facebook OR twitter OR internet OR web-based OR "mobile phone*" OR "cell phone*" OR texting OR "text messag*" OR smartphone* OR "mobile app" OR "mobile apps" OR "mobile application*" OR mhealth OR ehealth OR telehealth OR telemedicine OR multimedia OR “mass media”

AND

prevent*

AND

"substance abuse" OR "substance misuse" OR "substance dependence" OR "substance addiction" OR "substance use disorder*" OR "drug misuse" OR "drug abuse" OR "drug addiction" OR "drug dependence" OR "drug use disorder*" OR "alcohol abuse" OR "alcohol misuse" OR "alcohol dependence" OR "alcohol addiction" OR "alcohol use disorder*" OR alcoholism OR "tobacco abuse" OR "tobacco misuse" OR "tobacco dependence" OR "tobacco addiction" OR "tobacco use disorder*" OR "nicotine abuse" OR "nicotine misuse" OR "nicotine dependence" OR "nicotine addiction" OR "nicotine use disorder*" OR "smoking abuse" OR "smoking dependence" OR "smoking addiction"
