# Supplementary material for: Consumer Health Information Technology in the Prevention of Substance Abuse: Scoping Review
Source: J Med Internet Res. 2019 Jan 30;21(1):e11297. doi: 10.2196/11297 (PMC6372939; doi:10.2196/11297)
Supplement: Multimedia Appendix 2 [file jmir_v21i1e11297_app2.pdf]

Table 2: Summary of articles by study design, CHIT used, and outcomes

This is a Multimedia Appendix to a full manuscript published in the J Med Internet Res. For full copyright and citation information see http://doi.org/10.2196/preprints.11297

Title	Authors	Publication year	Study Design/Methodology (Study design used, N, Behavioral frameworks used if any, intervention if any)	CHIT used	Comments (Outcomes, conclusions, results, summary in brief)
Drug perception and the student-teacher gap. Reactions of 428 students and 72 teachers to an experimental trigger film on drugs [40]	Schuman, S. H.	1971	Cross-sectional survey data from open-ended questionnaire post film viewing, N - 428 students and 72 teachers, Field test of a film for teenagers emphasizing motivations of drug behavior rather than drug facts, chi-square	Film	The study found a large gap in drug perception by correcting identifying drug clues between faculty (12.5%) and students (32%). There was no significant difference in identification of drug clues by geographical or socioeconomic difference among schools. In order for drug educations to be effective, drug counselors and teachers need to understand subtle clues of drug use in ordinary, everyday context of students.
Drugs and the mass media [57][a]	Barcus, F. E.; Jankowski, S. M.	1975	Review of antidrug messages to understand the role of different mass media platforms in the field of drug use and abuse, results of 6 studies discussed	Mass media, non-specific	Mass media antidrug messages have been largely ineffective in changing behavior. One of the reasons is the media messages have not been tailored to reach specific segments of population by drug use status or age. The authors concluded the mass media alone is not sufficient to change behavioral changes. The media may serve as a source of information but decision to change behaviors is influenced by many other factors.
Attitudes toward alcohol and drug abuse. II. Experimental data, mass media research, and methodological considerations [47][a]	Kinder, B. N.	1975	Literature review of 14 studies, concerning relationship of demographic and personality variables to drug and alcohol-related attitudes.	Mass media (newspaper, magazine, radio and television	There are conflicting results about the effects of mass media on disseminating drug-related information. Drug users tend to rely on friends and their own experience as a source of drug information rather than media. In contrast, non-users are more likely to obtain drug information from mass media. However, no previous study results had demonstrated if the mass media are effective in changing an attitude toward drug use. Rather some professionals in the field believed fear-arousing tactics used by the mass media produced an undesirable effect of inducing young people to experiment with drugs. Overall, the mass media was shown to be ineffective in changing values and attitude that require strong commitment.
The critical assessment of the T.V. film "The Drug Takers" [30]	Milne, H. B.; Butt, T. W.	1975	Cross-sectional Survey before and after film viewing; Pre-test and post-test; N- 270 children (13-16 years of age), intervention - 20 minute film, material illustrated clinical facts and demonstrated relationships between the young drug abuser, society and the law, and in particular the young person's problems created by drug abuse; t-test	T.V, Film	No immediate change in knowledge and attitude was observed after viewing the film compared with before film viewing. The results suggested those who considered drug use is associated with social advantages also thought the dangers of drug use is exaggerated. Therefore, the authors suggested to explore advantages of being a non-drug user and presumed advantages of drug use as viewed by young people

Anti-drug abuse commercials [42]	Feingold, P. C.; Knapp, M. L.	1977	Survey after film viewing; junior and sophomore students in 10 classes; data collected in 6 sessions over 3 weeks from 60 students; T-test, ANOVA, correlation matrix, and principle components factor analysis	TV and radio	The message with explicit conclusion was shown to be more effective. However, there was no difference between monologue vs. dialogue or serious vs. minimal harm format. Contrary to the intention of the anti-drug message, many students changed their attitude toward drugs from negative to less negative. Due to the boomerang effect, the authors concluded anti-drug messages might lead adolescents to use drugs.
Mass media and drinking, smoking, and drug taking [56][a]	Wallack, L. M.	1980	Review to provide a history of mass media intervention for the prevention of drinking, smoking, and drug use, provided to the public and to make critical assessment of effectiveness of these interventions, results of 6 studies discussed	Mass media, non-specific	Though mass media campaign against smoking had little impact on changing behaviors of individual smokers, it did change the social structure in which smoking took place. Such that the pressure against smoking was far greater than for smoking. Anti-drinking and anti-drug did not bring about similar changes in social structure against abuse of these substances. The author advocated the use of monopolism (presence of no opposing views), canalization (directing existing views or behaviors in a similar but different direction), and supplementation (interpersonal contact in addition to mass media) for mass media campaign to be successful.
Comparison of media for substance abuse education in rural communities [41]	Miller, M. C., 3rd; Cantor, A. B.; Larisey, L.; Murphy, E.	1981	Cross-sectional survey after educational programs were offered, n=50; TV programs presented Saturday and Monday morning, and radio programs presented Sunday and Monday morning on2 different stations; chi-square used	TV, radio, newspaper	The study examined which mode of media is more effective in disseminating substance use education program to various age, racial groups, geographical areas, and income levels. The authors found radio to be not as effective as newspaper and TV. The study concluded in order for mass media campaign to be effective the program has to understand characteristics of target audience.
Mass media campaigns: the odds against finding behavior change [55][a]	Wallack, L. M.	1981	Review of the history and impact of large- scale campaigns for prevention of alcohol, drug, and tobacco abuse in communicating information to the general public. , results of 10 studies discussed.	Mass media, non-specific	Overall, mass media campaign has been shown to be ineffective to change behavior in consumption of substances. Some studies reported campaign against the use of drug and alcohol may have resulted in increased use of substances due to sensational effects of media messages. In general, the effectiveness of media is limited to increasing knowledge and reinforcing existing behavior. The author suggested the use of monopolization, canalization, and supplementation as conditions of media campaign success
Recent literature on drug abuse prevention and mass media: Focusing on youth, parents, women and the elderly [48][a]	Bandy, P.; President, P. A.	1983	Literature review of the mass media campaign and approaches to prevent drug abuse targeting four specific groups: youth, parents, women, and the elderly, results of 15 studies discussed	Mass media, non-specific	Of the four audiences, the youth target group has been studied the most in terms of drug abuse messages, credibility of sources, drug patterns, and so on. Parents play a dual role, as targets and as intermediaries. Although mass media efforts to reach parents have been minimal, this target group has a strong motivation for both receiving messages and for acting on them. Women have received attention primarily through the message of appropriate drug use. However, studies focused primarily on the channel as opposed to other elements of communication. The elderly are also targets of campaign messages on appropriate drug use. These efforts feature multiple media platforms, which frequently involve an intermediary interpreter. The importance of audience analysis, of segmenting audiences into subgroups, and of designing customized

					presentations has been cited as critical across all target populations.
The role of mass media in preventing adolescent substance abuse [54][a]	Flay, B. R.; Sobel, J. L.	1983	Review of classroom program or drug education using mass media and identify factors associated with successful program, results of 13 studies discussed	Mass media, non-specific	Most media prevention programs have failed. One reason may be it never reached the targeted audience. In addition, advertisement may influence behavioral changes if audiences are exposed to it three or more times. With drug prevention program, studies did not ensure the audience has watched the program multiple times. The messages themselves also have been either informational or fear arousing neither of which has been effective in changing behaviors. Effective mass media program should be able to gain the attention of audience and function as a supplement of other programs such as school-based curriculum or community organization intervention
The use of films in drug education--a review [27][a]	Pickens, K. A.	1984	Review of effectiveness of film in drug education compared with other media, 7 studies included	Film	The studies also reported film is not as effective as other types of media in brining on changes in knowledge, attitude, and behavior. Thus, authors concluded an intervention via film was not superior to non-film approaches. The short-term impact of film intervention did not last in long-term follow up in some studies. Measuring long-term effects would need to be assessed as well.
Mass media linkages with school-based programs for drug abuse prevention [43][a]	Flay, B. R.	1986	Literature review of 3 major papers on the role of media in the prevention of drug abuse using school-based interventions, results of 6 studies discussed	Non-specific	The study discussed critical components for successful media drug prevention program including overcoming apathy of audience and promoting discussion thru interpersonal communication. The authors emphasized importance of monopolization and canalization to improve effectiveness of media drug education. The intervention strategies using the media intervention should attempt to increase awareness of problems associated with substance abuse and to maintain positive attitude toward remaining drug-free. Furthermore, mass media is effective when it is used in conjunction with other types of intervention such as school-based programs to reinforce knowledge and skills learned in school and community programs.
Viewing and evaluation of a televised drug education program by students previously of concurrently exposed to school-based substance abuse prevention programming [37]	Sussman, S.; Flay, B. R.; Sobel, J. L.; Rauch, J. M.; Hansen, W. B.; Johnson, C. A	1987	Quasi-experimental study design, N - 2882 students (8th graders), survey after viewing TV program; intervention - PSA during prime-time viewing hours, 4 days of 5-min segments during early evening news hour discussing family, health consequences and other difficulties related to drug abuse, encouraging family involvement to prevent or stop drug abuse, and a half-hour interactive information program called 'The Drug Abuse Test'.	TV program	Those who received concurrent drug prevention program at school during the period the show aired were more likely to watch the show than the other two groups. The show seemed to have more positive impact when it was viewed with parents on learning, interest, and helpfulness. Being involved in previous school drug program did not affect the viewing of TV drug education program. As the frequency of viewing increased, students assessed the TV program more favorably. The authors concluded positive involvement of parents might improve the impact of mass media drug program.

Reactions to drug education: a comparison of two videos produced for schools [31]	Eiser, C.; Eiser, J. R.; Pritchard, M.	1988	Randomized control study design with post assessment questionnaire after film, N - 518 students (14 yo); social skills approach used; Intervention - 3 groups assessed, 2 films were reviewed, 'thinking twice' and 'minder'; control group viewed the minder film after the completion of questionnaires. Pre-test the first group was shown a video that was an entertaining story about how some teenagers became involved with drugs. The second group was shown a video that was more didactic and informational. The third group saw no video.	Video	Three groups of students were compared for effectiveness of drug education videos: an entertaining educational video about drug use, informational video, and no video viewing. The students who watched didactic video had increased knowledge about drug use whereas those who viewed entertaining video had more negative attitude toward drug use after viewing the video.
Reaching Hispanics with messages to prevent alcohol and other drug abuse [49][a]	Johnson, E. M.; Delgado, J. L.	1989	Review of survey data to identify at-risk Hispanic population and to describe strategies and efforts using media to reach this population, results of 4 studies discussed	Radio, TV	Based on previously published data, authors concluded there is a particular need for more research on communication programs to reach three groups of Hispanics: children and adolescents, women of childbearing age, and heavy-drinking men. Authors suggested due to the diversity of these groups of people, the contents of program must be developed in recognition of this diversity. Not much is known about attitude, social norms, values, and resistance behaviors toward alcohol and drug use in this population; understanding these factors would provide a solid base for program development. For each group of audience, a message that promotes positive changes in behaviors should capitalize on the value of familyism, a strong psychological factor among Hispanics.
Microcomputer-based approaches for preventing drug and alcohol abuse among adolescents from ethnic-racial minority backgrounds [53]	Moncher, M. S.; Parms, C. A.; Orlandi, M. A.; Schinke, S. P.; Miller, S. O.; Palleja, J.; Schinke, M. B.	1989	Quasi-experimental Posttest study, N- 26 11-14 years old black children from economically disadvantaged homes; Social cognition theory used; Intervention - demonstration program, 15 mins interaction with the software and brief description of learning task and basic keyboard use was presented. Questionnaire items measured contextual and interactional variables appropriate to the microcomputer task and software. Contextual variables measured in the battery included the amount of material retained, or learned, by subjects upon completion of computer interaction. These variables covered subjects' feelings about using computers respective to such factors as intimidation, mastery, enjoyment, and involvement.	Computer	Large percentage of participants (42%) did not have any objections to the use of computer to receive information. However, majority (69%) preferred to receive drug information or counseling delivered by human rather than computer

The television, school, and family project [36]	Brannon, B. R.; Dent, C. W.; Flay, B. R.; Smith, G.; Sussman, S.; Pentz, M. A.; Johnson, C. A.; Hansen, W. B.	1989	Posttest study, n=28, ANOVA	TV	TV format of intervention has a higher participation rates than classroom delivery format ($p<0.001$). Participants in TV format had a greater satisfaction with the program ($p<0.03$), but perceived program effectiveness was higher in combination of TV program and classroom program ($p <0.001$) than TV program alone. The authors concluded TV delivery format can be useful in wide implementation of program and enhancing effectiveness of classroom-based program.
Computer-delivered, parent-involvement intervention to prevent substance use among adolescent girls [44]	Schinke, S. P.; Fang, L.; Cole, K. C.	2009	Randomized control trial, N-916 girls age 11-13 years and their mothers, family interaction theory used; Intervention - web-based 0 9 sessions of 45 mins each and 1 annual booster session of 45 mins, Each session was delivered through voice-over narration, skills demonstrations by animated characters, and interactive exercises for mothers and daughters to complete jointly. Program exercises taught mothers and daughters the value of listening to each other, spending time together, understanding one another's personality, negotiating mutually agreeable decisions to problems, doing personal favors for one another, and giving each other praise and compliments	Internet, CD-ROM	The intervention group had lower relevant risk factors, higher protective factors, and less 30-day alcohol use ($p<0.006$), marijuana ($p<0.016$), illicit prescription drug use ($p<0.03$) and inhalants ($p<0.03$). Similar finding was seen among mothers in the intervention groups, there was less weekly use of alcohol ($p<0.0001$), Mothers who received the prevention program had better outcomes than control-arm mothers on variables associated with lower substance use risks for their adolescent daughters.
Using online components to facilitate program implementation: impact of technological enhancements to all stars on ease and quality of program delivery [45]	Hansen, W. B.; Bishop, D. C.; Bryant, K. S.	2009	Randomized field trial, 23 schools and 2 community groups, ANOVA, Chi-square	Web-based and CD ROM	Most teachers (83%) who used technology format of program preferred it to the standard format. However, more teachers using technology version made changes to activities in the program, though not statistically significant ($p=0.18$). Majority of students (>75%) agreed or strongly agreed online activities made an important contribution to the drug prevention program. The authors concluded school-based prevention program could benefit from adding web-based components to improve ease of implementation and enthusiasm of teachers
Preventing substance use among adolescent girls: 1-year outcomes of a computerized, mother-daughter program [51]	Schinke, S. P.; Fang, L.; Cole, K. C.	2009	Randomized control trial with N -591 mother-daughter pairs conducted; Family interaction theory was used for the intervention	Internet	The program showed positive effects on adolescent girls and their mothers one year after program delivery. Intervention-arm girls relative to control-arm girls improved on variables associated with lower risks for substance use, variables that can protect adolescents against future substance use, current use of alcohol, marijuana, and prescription drugs, and intentions to use tobacco, alcohol, and drugs in the future. Intervention-arm mothers uniformly benefited from the program across all of their measured outcome variables. Over time, girls in both arms increased their cigarette, alcohol, and

					marijuana use and their intentions to smoke, drink, and use illicit drugs in the future. However, the increases in cigarette, alcohol, and marijuana use for intervention-arm girls were more modest than those for control-arm girls. There was also less prescription use among intervention-group girls
Mass media interventions for preventing smoking in young people [28][a]	Brinn, M. P.; Carson, K. V.; Esterman, A. J.; Chang, A. B.; Smith, B. J.	2010	Systematic Review of 10 articles; Information relating to the characteristics and the content of media interventions, participants, outcomes, methods of the study and risk of bias was abstracted by two independent reviewers. Studies were combined using qualitative narrative synthesis. Most studies used the Social learning theory	Mass media (radio - TV)	Three out of seven studies demonstrated statistically and clinically significant reductions for smoking uptake in young people. Common features to these successful campaigns included multiple channels for media delivery (e.g. newspapers, television, radio, posters, etc.), combined school and media components (through school posters and/or school based curriculum), and repeated exposure to campaign messages consecutively delivered for the same cohort of students over a minimum period of three years. The other successful campaigns used provocative messages to cause effective personal reactions. However three of the remaining four studies, which did not produce any statistical benefit, also used the social influences approach. Two of the four unsuccessful studies had short campaign durations (two weeks for one study, and four weeks for another) and as such were less intense than the successful campaigns. The lack of a structured curriculum component to support these messages, such as those in the combined school based studies, likely accounts for the eventual failure in preventing the uptake of smoking in young people.
Preventing substance use among early Asian-American adolescent girls: initial evaluation of a web-based, mother-daughter program [34]	Fang, L.; Schinke, S. P.; Cole, K. C.	2010	Randomized control trial with N -108 Asian-American mother-daughter pairs conducted; Family interaction theory was used for the intervention; intervention - 9 session web-based substance use prevention program	Internet	At posttest, relative to control-arm girls, intervention-arm girls showed less depressed mood; reported improved self-efficacy and refusal skills; had higher levels of mother-daughter closeness, mother-daughter communication, and maternal monitoring; and reported more family rules against substance use. Intervention-arm girls also reported fewer instances of alcohol, marijuana, and illicit prescription drug use, and expressed lower intentions to use substances in the future.
Preventing drug abuse among adolescent girls: outcome data from an internet-based intervention [18]	Schwinn, T. M.; Schinke, S. P.; Noia, J.	2010	Randomized control trial; N-236 girls; Based on Social Learning Theory; Pre-Post design used; intervention - 12-session, internet based gender-specific drug prevention program	Internet	Adolescent girls who participated in the online intervention reported decreased 30-day alcohol, marijuana, poly drug, and total substance use at 6-month follow-up. Past-month cigarette use did not differ between groups. Posttest measures were completed immediately after intervention delivery. The trajectory of decreased substance use between posttest and follow-up for intervention-arm girls and increased use among control-arm girls suggests that girls may require time and real-life opportunities to apply acquired skills.

Short-term efficacy of Click City(R): Tobacco: changing etiological mechanisms related to the onset of tobacco use [58]	Andrews, J.A; Gordon, J. S.; Hampson, S. E.; Christiansen, S. M.; Gunn, B.; Slovic, P; Severson, H. H.	2011	Randomized Control trial; N - 2322; Multiple theories - Cognitive and risk perception theories; Intervention - 8 sessions in 5th grade and 2 booster sessions in 6th grade delivered over Intranet on school computers; Pre-post assessment	Intranet and Desktops	Results show that Click City®: Tobacco had the potential to postpone or prevent initiation of cigarette use. In addition, findings suggest that the program is efficacious at reducing all etiological risk factors associated with cigarette use, significantly lowering the risk of initiation. The program was particularly effective at decreasing intentions and willingness for those students who were particularly at risk, further adding to the efficacy of the program. It was only moderately effective at changing intentions to use smokeless tobacco in the future.
Preventing prescription drug misuse: field test of the SmartRx Web program [33]	Deitz, D. K.; Cook, R. F.; Hendrickson, A.	2011	Randomized control study; N-362; Social Cognitive theory was used; Intervention - web-based - using multimedia platforms like videos, graphics and interactive segments	Web-based (internet - includes video content)	Compared with the wait-list control group, users of the Web-based program significantly increased their knowledge of proper prescription drug use and had greater self-efficacy in their ability to manage and adhere to appropriate pharmaceutical treatment compared with the control group. In addition, the significant effects of the program on CAGE scores indicate that the Web-based SmartRx program resulted in a reduction of symptoms commonly associated with drug misuse problems.
Preventing substance use among Black and Hispanic adolescent girls: results from a computer-delivered, mother-daughter intervention approach [38]	Schinke, S. P.; Fang, L.; Cole, K. C.; Cohen Cutler, S.	2011	Randomized control study; N-546 mother daughter pairs; family interaction theory used; intervention - web based 10 sessions - session included voice-over narration, skills demonstrations by animated characters, and interactive exercises for mothers and daughters to complete jointly, sessions imparted gender-specific content and skills to girls and their mothers toward helping the girls reduce salient risk factors and build protective factors associated with successful avoidance of tobacco, alcohol, and drug use during the teen years.; pre-post assessment	Internet or CD-ROM	Following the program delivery, girls showed improvements in their communication with their mothers, in their understanding of family rules regarding substance use, and in their awareness of parental monitoring of their social activities and friendships. Further, girls improved their normative beliefs about the extent of substance use among their peers, were less depressed, and expressed higher levels of self-efficacy regarding their ability to not smoke, drink, and use drugs. The program also resulted in lower reports of girls' alcohol use and in their lower expectations of future tobacco, alcohol, and prescription drug use. Mothers who took part in the program improved their communication with their daughters, reported more family rules against substance use.
Potential exposure to anti-drug advertising and drug-related attitudes, beliefs, and behaviors among United States youth, 1995-2006 [35]	Terry-McElrath, Y. M.; Emery, S.; Szczypka, G.; Johnston, L. D.	2011	Cross-sectional study; N - 332586 8th, 10th and 12th grade students; exposure to anti-drug television commercials were tracked and measured using the Nielson Media research ratings and were correlated with the data obtained from the 'Monitoring the Future Study' on drug related outcomes from 1995-2006	TV ads	American youth were clearly aware of the anti-drug advertising occurring in the media around them. Two-thirds of all youth reported seeing anti-drug ads on TV or hearing them on the radio at least weekly in recent months, and the odds of such recall were significantly and positively related to total and marijuana-focused anti-drug advertising. Marijuana-focused advertising appeared to be effective among middle school youth, with indications that such advertising with the combined ONDCP/PDFA tagline was especially relevant. In contrast, high school youth presented a very different set of relationships. The current analysis demonstrates no global statement can be made about the efficacy of using televised anti-drug media campaigns to combat substance use among youth. Putting a concerted effort into increasing anti-drug advertising will likely increase the exposure to and recall of such ads among youth. However, the likelihood that such advertising will have positive impact on youth seems to depend

					heavily on the type of advertising utilized and how it relates to different ages and characteristics of targeted youth.
A systematic review of school-based alcohol and other drug prevention programs facilitated by computers or the internet [21][a]	Champion, K. E.; Newton, N. C.; Barrett, E. L.; Teesson, M.	2013	Systematic Review of 12 articles; Studies were included if they described an Internet- or computer-based prevention program for alcohol or other drugs delivered in schools, programs targeting school aged students that were implemented in the home or community were excluded, as were those delivered to university or college students. Study quality was evaluated using a validated tool for assessing the quality of randomized controlled trials; Most studies used the Social influence theory	Internet and CD-ROM	Six of the seven programs achieved a reduction in alcohol or drug use at post intervention and/or follow up, two were associated with decreased intentions to smoke in the future and two programs significantly increased alcohol- and drug-related knowledge. The results of the present review indicate that existing computer- and Internet-based programs in schools are a potentially efficacious method of delivering drug and alcohol pre-venation to adolescents. The greatest effects were achieved in relation to drug- and alcohol-related knowledge; with effectiveness persisting at 6- and12-month follow ups for three trials.
The impact of science education games on prescription drug abuse attitudes among teens: a case study [39]	Klisch, Y.; Bowling, K. G.; Miller, L. M.; Ramos, M. A.	2013	Quasi-experimental study (pre-post assessment); N - 179 students from 11th and 12th grade; theory of reasoned action was used; intervention - 4 web-based interactive game sessions offered in school to increase knowledge about opioid pain reliever abuse and drug interactions	Internet	The results presented in this suggest that including science and health-related information about prescription drug abuse as a subtext to forensic science can be effective in promoting healthier attitudes toward non-medical use of prescription drugs. Student's demonstrated shifts toward more negative prescription drug abuse attitudes observed in both study groups, resulting from students interacting with their assigned game in two class periods, reflected a moderate effect size for Bitter Pill and a large effect size for Fatal Interactions. Overall, students in both groups rated their satisfaction with the games and their level of engagement while playing the games above average.
Long-term efficacy of click city: tobacco: a school-based tobacco prevention program [46]	Andrews, J. A.; Gordon, J. S.; Hampson, S. H.; Gunn, B.; Christiansen, S. M.; Slovic, P.	2014	Randomized control trial design; N - 2232 5th grade students; Social cognition and risk perception theories used; intervention - interactive computer based program delivered in school	Internet and school desktops	Results show Click City ®: Tobacco may have the potential to postpone or prevent initiation of cigarette use and regular smoking. In addition, findings suggest that the program was efficacious at reducing several etiological risk factors associated with cigarette use, which may lower the risk of initiation. However, there was long-term effect was not observed in the study. The positive effects shown in the study, however, could be due to low intentions to chew tobacco among these young children across both conditions.
Universal Internet-based prevention for alcohol and cannabis use reduces truancy, psychological distress and moral disengagement: a cluster randomized controlled trial [22]	Newton, N. C.; Andrews, G.; Champion, K. E.; Teesson, M.	2014	Randomized control trial design; N - 764 13-14 year old students; Social influence approach in harm minimization framework used; intervention - climate Schools: Alcohol and cannabis course delivered in 2 sets of six 40 min sessions aimed at decreasing alcohol misuse and cannabis use.	Internet	Results indicate that compared to the control group, students in the intervention group showed significant reductions in truancy, psychological distress and moral disengagement up to twelve months following completion of the intervention. Findings from this study demonstrate for the first time that an Internet-based preventive intervention, which has been found to decrease substance use, can concurrently reduce associated risk factors in adolescents. Schools should be encouraged to implement evidence-based programs within their curriculum to alleviate the significant burden of disease, harms and social costs associated with adolescent substance use.

Computer-based programs for the prevention and management of illicit recreational drug use: a systematic review [19][a]	Wood, S. K.; Eckley, L.; Hughes, K.; Hardcastle, K. A.; Bellis, M. A.; Schrooten, J.; Demetrovics, Z.; Voorham, L.	2014	Systematic Review of 10 articles; the study evaluates a computer-based intervention for preventing, reducing or managing illicit recreational drug use; Studies were included if they described a computer-based prevention program. Study quality was evaluated independently by two reviewers using a validated tool for assessing the quality of randomized controlled trials; Most studies used the Social influence theory	Computer	This review suggests that computer-based programs have the potential for use in addressing illicit recreational drug use when targeted at both universal populations and illicit drug users. However, more research is needed to establish long-term effectiveness (> 12 months) and explore the use of programs outside of English speaking countries.
Reducing substance involvement in college students: a three-arm parallel-group randomized controlled trial of a computer-based intervention [25]	Christoff Ade, O.; Boerngen-Lacerda, R.	2015	Randomized control trial; N - 815 >= 18 years undergraduate students; motivational interview technique used; intervention-ASSISTi and ASSISTc	Computer	A detailed analysis showed that, for alcohol, the computer-based intervention reduced specific scores compared with the control group and the two formats reduced the scores for each question at follow-up. For marijuana, a small positive effect was observed at follow-up in the interview and control groups, suggesting low effectiveness. For tobacco and other drugs, despite the decrease in specific involvement scores in the three groups at follow-up, inconsistency was observed within groups in the scores for each question, and no significant difference was observed compared with the control group.
The effectiveness of electronic approaches to substance abuse prevention for adolescents [20][a]	Hopson, L.; Wodarski, J.; Tang, N.	2015	Systematic Review of 28 articles; the study evaluates the effectiveness of electronic interventions using computers or web-based platforms (internet, CD-ROM or videos) for preventing use of substances, including alcohol, tobacco, or illicit drugs, or aimed to reduce risk for substance use. Studies were included if they described an computer-based prevention program and included a control or comparison condition	Internet, video and CD-ROM	Results show that computer- and Web-based approaches provide a cost-effective option for reaching more individuals, including those who have limited access to service providers. The present review also demonstrates that many computer- and web-based interventions have solid research support. Although most of the studies included in this review demonstrated positive effects of electronic interventions, some evaluations indicate that computer- and Web-based prevention have limited or no effects relative to interventions delivered using traditional methods.
A cross-validation trial of an Internet-based prevention program for alcohol and cannabis: Preliminary results from a cluster randomized controlled trial [23]	Champion, K. E.; Newton, N. C.; Stapinski, L.; Slade, T.; Barrett, E. L.; Teesson, M.	2016	Randomized control trial design; N - 1103 13-14 year old students; Social influence approach in harm minimization framework used; intervention - climate Schools: Alcohol and cannabis course delivered in 2 sets of six 40 min sessions aimed at decreasing alcohol misuse and cannabis use.	Internet	Results indicate that the program was effective in increasing alcohol and cannabis knowledge, and there was some evidence that the intervention reduced students' intentions to use alcohol and delayed the use of any alcohol (including even a sip or taste). However, the module did not have an impact on binge drinking, cannabis use in the past 6 months or intentions to use cannabis. The program showed significant positive effect on the intention to use alcohol and no significant effect on the intention to use cannabis. There were no significant intervention effects for binge drinking and cannabis use at post-intervention, which is consistent with the results from the original trial of the Climate Schools course
Effectiveness of a universal	Champion, K. E.;	2016	Randomized control trial design; N - 1126 14-15 year old	Internet	Students in the control group were more than 10 times as likely to use NPS and more than three

internet-based prevention program for ecstasy and new psychoactive substances: a cluster randomized controlled trial [24]	Newton, N. C.; Stapinski, L. A.; Teesson, M.		students; Social influence approach in harm minimization framework used; intervention - climate Schools: ecstasy and emerging drugs module delivered in four 20 min sessions aimed to impart evidence-based information about ecstasy and NPS, correct overestimates of peer ecstasy and NPS use, teach refusal skills and address misconceptions.		times as likely to intend on using synthetic cannabis at the 12-month follow-up. Students in the control group also reported significantly less knowledge about both NPS and ecstasy at post-test. However, there were no intervention effects for actual use of ecstasy and NPS up to 12 months following the intervention. Evaluation and fidelity data, combined with changes in intentions and knowledge, suggest that it is possible to deliver online prevention for NPS and ecstasy simultaneously. The intervention may be of particular use to international populations that are experiencing problematic rates of youth NPS and ecstasy use. Adaptation of previous Climate Schools programs for an international context.
Prevention of alcohol and other drug use and related harm in the digital age: what does the evidence tell us? [26][a]	Champion, K. E.; Newton, N. C.; Teesson, M.	2016	Systematic Review of 12 articles; the focus of this review is on preventing the onset and delaying the uptake of alcohol and/or drug use using online or computer based interventions among school aged children (11-18 year olds). Hence, RCT's, which specifically evaluated this age group and intervention modalities, only were included in the review.	Computer and internet (online platforms)	In order for the online interventions to advance toward widespread dissemination, further replication and longer-term trials are needed to better understand the effectiveness of interventions and to examine effects at both a symptom and disorder level. Attempts to increase impact are also required, which could be achieved by targeting additional agents of change, such as parents, or addressing additional behavioral risk factors alongside alcohol and other drugs. Despite these issues, there is clear evidence that online prevention for alcohol and other drugs can be effective.
Mass media interventions for preventing smoking in young people [29][a]	Carson, K. V.; Ameer, F.; Sayehmiri, K; Hnin, K.; Vanagteren, J. E.; Sayehmiri, F.; Brinn, M. P.; Esterman, A. J.; Chang, A. B.; Smith, B. J.	2017	Systematic Review of 8 articles; Information relating to the characteristics and the content of media interventions, participants, outcomes, methods of the study and risk of bias was abstracted by two independent reviewers. Studies were combined using qualitative narrative synthesis. Most studies used the Social learning theory. The majority of studies focused on young adults aged <25 years of age	Social media, TV, radio, mass media	Three out of eight studies found that the intervention was effective in preventing smoking in youth. The remaining five studies did not detect an effect. Although there was some overlap in characteristics between both effective and ineffective programs, effective campaigns tended to last longer (minimum 3 years) and were more intense (more contact time) for both school-based lessons (minimum eight lessons per grade) and media spots (minimum four weeks' duration across multiple media channels with between 167 and 350 TV and radio spots). Implementation of combined school-based components (e.g. school posters) and the use of repetitive media messages delivered by multiple channels (e.g. newspapers, radio, television) appeared to contribute to successful campaigns.
A Mobile Phone-Based Life Skills Training Program for Substance Use Prevention Among Adolescents: Pre-Post Study on the Acceptance and Potential Effectiveness of	Haug, S.; Paz Castro, R.; Meyer, C.; Filler, A.; Kowatsch, T.; Schaub, M. P.	2017	Quasi-experimental study (pre-post assessment); N - 1067 school going students; social cognitive theory was used; intervention - The fully automated program, named ready4life was used. It addressed self-management skills, social skills, and substance use resistance skills. Program participants received up to 3 weekly text messages (short message service, SMS) over 6 months. Active program engagement was stimulated by	Mobile	The study revealed three main findings: (1) a large proportion of the eligible adolescents who were invited for program and study participation in the setting of a school classroom, participated; (2) the majority of program participants completed the entire program and engaged in program activities; however, regular program use could be improved; and (3) the initial results derived from this pre-post comparison revealed statistically significant increases in the life skills addressed, a decline in at-risk alcohol use, and stable prevalence rates for tobacco and cannabis use. The proportion of adolescents with at-risk alcohol use was reduced by a quarter from baseline assessment to follow-up,

the Program, Ready4life [32]			interactive features such as quiz questions, message- and picture-contests, and integration of a friendly competition with prizes in which program users collected credits with each interaction.		whereas no significant changes were obtained in the prevalence of tobacco and cannabis use.
Beyond face-to-face individual counseling: A systematic review on alternative modes of motivational interviewing in substance abuse treatment and prevention [50][a]	Jiang, S.; Wu, L.; Gao, X.	2017	Systematic Review of 25 articles; Randomized clinical trials (RCTs) that evaluated the effectiveness of alternative modes of MI (other than face-to-face individual counseling) in preventing and treating substance abuse were included. Eligible studies were rated on methodological quality and their findings were qualitatively synthesized using the CONSORT 2010 guidelines	Telephone, internet communications, mobiles (SMS)	Beyond face-to-face counseling, telephone was the most frequently used medium for delivering MI (11 studies), followed by Internet communication (4 studies) and short message service (SMS) (2 studies). Mail was incorporated as a supplement in one of the studies for telephone MI. The effectiveness of telephone MI in treating substance abuse was supported by all of the published RCTs included. Collectively, the studies reviewed indicate that telephone MI is a promising mode of intervention in treating and preventing substance abuse. The effectiveness of other alternative modes (SMS-based MI, Internet-based MI and group MI) remains inconclusive given the controversial findings and a limited number of studies. By synthesizing the currently available evidence, this systematic review suggested that telephone MI might be considered as an alternative to face-to-face MI for treating and preventing substance abuse.
A Systematic Review of the mHealth Interventions to Prevent Alcohol and Substance Abuse [59][a]	Kazemi, D. M.; Borsari, B.; Levine, M. J.; Li, S.; Lamberson, K. A.; Matta, L. A.	2017	Systematic Review of 12 articles; Mobile intervention for substance use literature from 2005-2015 was searched and included. Studies were excluded if they used computer web-based interventions exclusively for screening. Data on study design, sample, intervention, results and outcomes was collected.	Mobile	Although various primary outcomes were studied, most of these papers found at least partial positives results. Although mHealth interventions can reach a large number of individuals and promote self-regulation, there is a need for larger sample sizes and longitudinal studies in future investigations of mHealth interventions for substance use, as larger sample sizes would help increase generalizability, and longitudinal studies would help researchers understand the long-term effects of the interventions. The current review supports the mounting evidence that mHealth technology is a promising means to address substance use and warrants further development and study. Given the familiarity and comfort that young adults have with mobile phones and their willingness to adopt new trends, there is an exciting opportunity to use mHealth interventions to reduce substance use either by themselves or through enhancing traditional intervention techniques by increasing access to real-life contexts within one's natural environments.

An Online Drug Abuse Prevention Program for Adolescent Girls: Posttest and 1-Year Outcomes [52]	Schwinn, T. M.; Schinke, S. P.; Hopkins, J.; Keller, B.; Liu, X.	2017	Randomized control trial design; N - 788 13-14 year old girls; Social learning theory used; intervention - Real teen - Online program, comprised of two components: the homepage and the intervention sessions. The homepage for the program was accessible at any time, included feeds from the latest entertainment sites, online polls, horoscopes, beauty tips, and a quote of the day. The nine intervention sessions (15-20 mins each) focused on goal setting, decision-making, puberty, body image, coping, drug knowledge, refusal skills (two sessions), and a review.	Internet and social media	The intervention program tested in this study produced material changes not only in girls' drug use behavior, but also in cognitions and skills that are empirically linked to reduced drug use risks. These include higher self-esteem, positive goal setting, increased media literacy, and drug refusal self-efficacy. As expected of any successful drug abuse prevention program, the intervention positively impacted girls' drug use behavior, including their cigarette use and binge drinking. That the positive impacts of the intervention were also seen in rates of peer drug use suggests that the program had a beneficial effect on girls' friendship networks. Continued positive outcomes at 1-year follow-up serve to underscore the social-emotional learning that apparently resulted in girls' participation in the intervention.

[a] Compilation of citations of studies included in this review is provided in Multimedia Appendix 3.

[a] Compilation of citations of studies included in this review is provided in Appendix B.
