# Supplementary material for: Consumer Health Information Technology in the Prevention of Substance Abuse: Scoping Review
Source: J Med Internet Res. 2019 Jan 30;21(1):e11297. doi: 10.2196/11297 (PMC6372939; doi:10.2196/11297)
Supplement: Multimedia Appendix 3 [file jmir_v21i1e11297_app3.pdf]

Multimedia Appendix: Studies included in the reviews cites in the scoping review

This is a Multimedia Appendix to a full manuscript published in the J Med Internet Res. For full copyright and citation information see http://doi.org/10.2196/preprints.11297

Title	Authors	Year of publicat-ion	Number of studies	Studies
Drugs and the mass media [57]	Barcus, F. E.; Jankowski, S. M.	1975	4	1. Kanter DL. THE DRUG SCENE: CURRENT RESEARCH. Public opinion quarterly; 1971;35(3):459-63.[75]<br>2. O'Keefe MT. The Anti-Smoking Commercials: A Study of Television's Impact on Behavior. The Public Opinion Quarterly; 1971;35(2):242-8.[76]<br>3. Fejer D, Smart RG, Whitehead PC, Laforest L. Sources of Information About Drugs Among High School Students. The Public Opinion Quarterly; 1971;35(2):235-41. [77]<br>4. Hanneman GJ. Sources of Drug Abuse Information on the College Campus. University of Connecticut; 1972 Contract No.: Report. [78]
Attitudes toward alcohol and drug abuse. II. Experimental data, mass media research, and methodological considerations [47]	Kinder, B. N.	1975	6	1. Linsky AS. The changing public views of alcoholism. Quarterly journal of studies on alcohol; 1970;31(3):692-704.[79]<br>2. Lipp MR, Benson SG, Taintor Z. Marijuana Use by Medical Students. AJP. 1971;128(2):207-12. doi: 10.1176/ajp.128.2.207; 0110.1176/ajp.128.2.207.[80]<br>3. Pollock SH. Attitudes of Medical Students Toward Marijuana. Journal of psychedelic drugs; 1972;5(1):56-61. doi: 10.1080/02791072.1972.10471470.[81]4. Fejer D, Smart RG, Whitehead PC, Laforest L. Sources of Information About Drugs Among High School Students. The Public Opinion Quarterly; 1971;35(2):235-41.[77]<br>5. Grant JA. DRUG EDUCATION BASED ON A KNOWLEDGE, ATTITUDE, AND EXPERIENCE STUDY. Journal of School Health; 1971;41(7):383-6. doi: 10.1111/j.1746-1561.1971.tb04438.x; 01.[82]<br>6. Zajonc RB. The Attitudinal Effects of Mere Exposure. Journal of Personality and Social Psychology; 1968;9. doi: 10.1037/h0025848. [83]<br>7. Amendolara FR. Modifying Attitudes towards Drugs in Seventh Grade Students. Journal of drug education; 1973;3(1):71-8. doi: 10.2190/WL56-DEV7-441M-LDDW; 05[84]<br>8. Braxton ER, Yonker RJ. Does being urban, poor, black, or female affect youth's knowledge and-or attitudes relating to drugs? The Journal of school health; 1973;43(3):185-8. PMID: 4486608. Language: English. Date Revised: 20041117. Date Created: 19730301. Date Completed: 19730416. Update Code: 20171127. Publication Type: Journal Article. Journal ID: 0376370. Publication Model: Print. Cited Medium: Print. NLM ISO Abbr: J Sch Health. Linking ISSN: 00224391. Subset: IM.[85]<br>9. Einstein S, Lavehar M, Garitano WW. Drug abuse education and the multiplier effect: an experience in training 109 teachers. The Journal of school health; 1972;42(10):609-13. PMID: 4485223[86]<br>10. Einstein S, Others A. The Training of Teachers for Drug Abuse Education Programs: Preliminary Considerations. Journal of Drug Education; 1971.[87]<br>11.Fau RDW, Fau NPR, Fau RKJ, Friedman SB. Attitudes of fifth grade students to illicit psychoactive drugs: J Sch Health; 1972 Sep;42(7):389-91; ISBN: 0022-4391.[88]<br>12.Irwin RP, Creswell WH, Stauffer DJ. The Effect of the Teacher and Three Different Classroom Approaches on Seventh Grade Students˜ Knowledge, Attitudes and Beliefs About Smoking*. Journal of School Health. 1970;40(7):355-9. doi: 10.1111/j.1746-1561.1970.tb05622.x; 05[89]<br>13. Levitt L, Baganz P, Blachly P. A study of employee attitudes toward patients in a hospital for the treatment of drug addiction. Psychiatric Quarterly. 1963;37(2):210.[90]<br>14. Shaw CT. Knowledge and Attitude Responses of College Students toward Controversial Social Health Issues. Journal of School Health. 1972;42(1):53.[91]

Mass media and drinking, smoking, and drug taking [56]	Wallack, L. M.	1980	6	1. Barcus F. Drug advertising on television in drug use in America: problem in perspective. Washington,DC: US goverment printing office; 1973 Contract No.: Report. [92]<br>2. Hanneman G, Eisenstock BHM, Weinbeck W. The medicine man message: an evaluation of a California state office of narcotics and drug abuse prevention campaign to inform the public of the dangers of prescription and over-the -counter drug misuse. University of Southern California, Pacific Institute for Research and Evaluation; 1977 Contract No.: Report.[93]<br>3. Kinder BN. Attitudes toward alcohol and drug abuse. II. Experimental data, mass media research, and methodological considerations. The International journal of the addictions; 1975;10(6):1035-54. doi: 10.3109/10826087509028359.[59]<br>4. O'Keefe MT. The Anti-Smoking Commercials: A Study of Television's Impact on Behavior. The Public Opinion Quarterly; 1971;35(2):242-8. [76]<br>5. Warner KE. The effects of the anti-smoking campaign on cigarette consumption. American journal of public health; 1977;67(7):645-50.*[94]*<br>6. Wotring C, Heald G, Carpenter C. Evaluation of the Florida drug abuse campaign (1976-1977). Florida state university: college of communication; 1977 Contract No.: Report.[95]
Mass Media Campaigns: The Odds Against Finding Behavior Change.	Wallack, L. M.	1981	10	1. Wallack L. An assessment of drinking patterns, problems., knowledge and attidtudes in three Northern California communities. Social research group; 1978 Contract No.: Report.[96]<br>2. Hanneman G, Eisenstock BHM, Weinbeck W. The medicine man message: an evaluation of a California state office of narcotics and drug abuse prevention campaign to inform the public of the dangers of prescription and over-the -counter drug misuse. University of Southern California, Pacific Institute for Research and Evaluation; 1977 Contract No.: Report. [93]<br>3. Brecher E. Licit and illicit drugs: Brown and company; 1972.[97]<br>4. Kinder BN. Attitudes toward alcohol and drug abuse. II. Experimental data, mass media research, and methodological considerations. The International journal of the addictions; 1975;10(6):1035-54. doi: 10.3109/10826087509028359.[59]<br>5. Lazarsfeld P, Merton R. Mass communication, popular taste, and organized social aciton. In: Schramm W, editor. The commnicaiton of ideas. 2 ed: Institute for religious and social studies; 1975.[98]<br>6. O'Keefe MT. The Anti-Smoking Commercials: A Study of Television's Impact on Behavior. The Public Opinion Quarterly; 1971;35(2):242-8.[76]<br>7. Warner KE. The effects of the anti-smoking campaign on cigarette consumption. American journal of public health; 1977;67(7):645-50.[94]<br>8. Fishbein F. Consumer beliefs and behavior with respect to ciagrette smoking. A critical analysis of the public literature. Federal Trade Commission; 1977 Contract No.: Report. [99]<br>9. Long range health planning b, non-medical use of d. Smoking and health in Canada. Department of national health and welfare; 1977 Contract No.: Report.[100]<br>10. Wotring C, Heald G, Carpenter C. Evaluation of the Florida drug abuse campaign (1976-1977). Florida state university: college of communication; 1977 Contract No.: Report. Mosher, J. F., & Wallack, L. M. (1979). *Proposed reforms in the regulation of alcoholic beverage advertising article]* Retrieved from http://survey.hshsl.umaryland.edu/?url=http://search.ebscohost.com.proxy-hs.researchport.umd.edu/login.aspx?direct=true&db=edshol&AN=edshol.hein.journals.condp8.13&site=eds-live [95]

Recent literature on drug abuse prevention and mass media: Focusing on youth, parents, women and the elderly [48]	Bandy, P.;President, P. A.	1983	15	1. Hanneman GJ, McEwen WJ. Televised Drug Abuse Appeals: A Content Analysis. Journalism & Mass Communication Quarterly - JOURNALISM MASS COMMUN; 1973;50:329-33. doi: 10.1177/107769907305000216. [101]<br>2. Schlegel RP. The Role of Persuasive Communications in Drug Dissuasion. Journal of drug education; 1977;7(3):279-90. doi: 10.2190/XWTJ-X7CT-0PGD-Y70E; 01<br>10.2190/XWTJ-X7CT-0PGD-Y70E. [102]<br>3. Dembo R, Schmeidler J, Upton DS, Babst DV, Diamond SC, Spielman CR, et al. A Survey of Students' Awareness of and Attitudes toward Drug Abuse Prevention Programs in New York State, Winter 1974/75. International Journal of the Addictions; 1979;14(3):311.[103]<br>4. Kline JA. Evaluation of a Multimedia Drug Education Program. Journal of Drug Education; 1972.[104]<br>5. Dembo R, Miran M, Babst DV, Schmeidler J. The believability of the media as sources of information on drugs. The International journal of the addictions; 1977;12(7):959-69.[105]<br>6. Smart RG, Fejer D. Credibility of Sources of Drug Information for High School Students. Journal of Drug Issues; 1972;2(2):8-18. doi: 10.1177/002204267200200204; 01<br>10.1177/002204267200200204.[106]<br>7. Feingold PC, Knapp ML. Anti-drug abuse commercials. The Journal of communication; 1977;27(1):20-8.<br>[26]<br>8. Milavsky JR, Pekowsky B, Stipp H. TV DRUG ADVERTISING AND PROPRIETARY AND ILLICIT DRUG USE AMONG TEENAGE BOYS*. Public opinion quarterly; 1975;39(4):457-81.<br>[107]<br>9. Payne DE. The Relationship between Television Advertising and Drug Abuse among Youth: Fancy and Fact. Journal of drug education; 1976;6(3):215-20. doi: 10.2190/R52G-U7R1-0N0D-DWCM; 01.<br>[108]<br>10. Kohn PM, Snook S. Balanced vs. One-sided Communications About Drugs. Journal of Drug Education; 1976.[109]<br>11. Wotring CE, Others A. Attacking the Drug Norm: Effects of the 1976-77 Florida Drug Abuse TV Campaign. Journal of drug education; 1979;9(3):255-61. PMID: EJ209226.[110]<br>12. Schmeling DG, Wotring CE. Agenda-Setting Effects of Drug Abuse Public Service Ads. Journalism Quarterly; 1976;53(4):743-6. doi: 10.1177/107769907605300424; 01.<br>[111]<br>13. Hanneman G, et al. The medicine man message: a delayed effects evaluation of a California state office of narcotics and drug abuse prevention campaign to inform the public of the dangers of prescription and over-the -counter drug misuse. University of Southern California, Center for communications policy research; 1978 Contract No.: Report.<br>[112]<br>14. Hanneman G, Eisenstock BHM, Weinbeck W. The medicine man message: an evaluation of a California state office of narcotics and drug abuse prevention campaign to inform the public of the dangers of prescription and over-the -counter drug misuse. University of Southern California, Pacific Institute for Research and Evaluation; 1977 Contract No.: Report. [93]<br>15. Sandmaier M. Myths and messages: using the media as a prevention tool, in the women next door: summary proceedings of a symposium on the subject of drugs and the modern women. US journal of drug and alcohol dependence; 1980. *[113]*

The role of mass media in preventing adolescent substance abuse [54]	Flay, B. R.;Sobel, J. L.	1983	13	1. Capalaces R, Starr J. The negative message of anti-drug spots: does it get across? Public telecommunications review; 1973;1:64-6. [114]<br>2.Delaney R. Florida study looks at effects of media messages. NIAAA information and feature service; 1981 Contract No.: Report. [115]<br>3Dickman FB, Keil TJ. Public television and public health. The case of alcoholism. Journal of studies on alcohol; 1977;38(3):584-92. [116]<br>4.Field T, Deitrick S, Hersey J, Probst J, Theologus G. Implementing public education campaigns:lessons from alcohol abuse prevention. Summary report to NIAAA. washington,DC: Kappa systems; 1983 Contract No.: Report.[117]<br>5.Hanneman GJ, McEwen WJ, Coyne SA. Public service advertising on television. Journal of Broadcasting; 1973;17(4):387-404. doi: 10.1080/08838157309363703.[118]<br>6. Hanneman G, et al. The medicine man message: a delayed effects evaluation of a California state office of narcotics and drug abuse prevention campaign to inform the public of the dangers of prescription and over-the -counter drug misuse. University of Southern California, Center for communications policy research; 1978 Contract No.: Report.[112]<br>7. Kinder BN. Attitudes toward alcohol and drug abuse. II. Experimental data, mass media research, and methodological considerations. The International journal of the addictions; 1975;10(6):1035-54. doi: 10.3109/10826087509028359.[59]<br>8. Morrison A, Kline F, Miller P. Aspects of adolescent information acquisition about drugs and alcohol topics. Ostman R, editor. London: Sage publication; 1976.[119]<br>9. O'Keefe MT. The Anti-Smoking Commercials: A Study of Television's Impact on Behavior. The Public Opinion Quarterly; 1971;35(2):242-8.[76]<br>10. Plant MA, Pirie F, Kreitman N. Evaluation of the Scottish Health Education Unit's 1976 campaign on alcoholism. Social psychiatry; 1979;14(1):11-24. doi: 10.1007/BF00583569.<br>[120]<br>11. Rappeport M, Labow PWJ. The public evaluates the NIAAA public education campaign: a sudy for the U.S. department of health, education, welfare, public health service, alcohol, drug abuse, and mental health administration. Princeton, New Jersey: Opinion research corporation; 1975.[121]<br>12.Trager R. Adolescent reactions to educational media messages regarding drug education. In: Ostman R, editor. Communication research and drug education: Sage publications; 1976.[122]<br>13.Wong MR, Barbatsis GS. Attitude and Information Change Effected by Drug Education via Broadcast Television and Group Viewing; 1976 Contract No.: Report.[123]

The use of films in drug education--a review [27]	Pickens, K. A.	1984	7	1. Trager R. Adolescent reactions to educational media messages regarding drug education. In: Ostman R, editor. Communication research and drug education: Sage publications; 1976.[122]<br>2. Weimer J. The effects of film treatments on attidutdes that correlated with drug-behavior: University of South Dakota; 1976.[124]<br>3.English GE. THE EFFECTIVENESS OF EMOTIONAL-APPEAL VERSUS FACT-GIVING DRUG EDUCATIONAL FILMS. Journal of School Health; 1972;42(9):540-1. doi: 10.1111/j.1746-1561.1972.tb00975.x; 03.[125]<br>4.Swift B. Evaluation of drug education : findings of a national research study of effects on secondary school students of five types of lessons given by teachers. Institute for the study of drug dependence; 1974 Contract No.: Report.[126]<br>5. Thornell J. The construction and evalution of drug education of a drug education programme for third grade students: Boston College; 1976.[127]<br>6.Sohn M. Change in factual knowledge and reported use of illicit drugs resulting from the viewing of a motion picture: University of Maryland; 1976.[128]<br>7.Taussig W. The effects of a family life program and a drug education program on the self esteem of fifth grade children: New York University; 1978.[129]
Mass media linkages with school-based programs for drug abuse prevention [43]	Flay, B. R.	1986	6	1. Rosengren K, Wenner L, Palmgren P. Media gratifications research: current perspective: Sage; 1985.[130]<br>2.Flay B, Pentz M, Johnson C, Sussman S. Reaching children with mass media health promotion programs: the relative effectivenss of an advertising campaign, a community-based program and a school-based program. In: Leather G, editor. Health evaluatoin and the media: Pergamon; 1986.[131]<br>3.Flay BR. Psychosocial approaches to smoking prevention: a review of findings. Health psychology : official journal of the Division of Health Psychology, American Psychological Association; 1985;4(5):449-88.[132]<br>4.Flay BR. Mass media and smoking cessation: a critical review. American journal of public health; 1987;77(2):153-60.[122]<br>6. Dickman FB, Keil TJ. Public television and public health. The case of alcoholism. Journal of studies on alcohol; 1977;38(3):584-92.[116]

Reaching Hispanics with messages to prevent alcohol and other drug abuse [49]	Johnson, E. M.; Delgado, J. L.	1989	4	1. National Coalition of Hispanic H, Huamn Services O. Early intervention with Hispanic youth. Washington,DC: Office for Substance Abuse Prevention, Alcohol, Drug Abuse and Mental Health Administration. (COSSMHO); 1988 Contract No.: Report.[134]<br>2.Amaro H, Campa R, Coffman G, Heeren T. Initiation of substance abuse among Mexican American, Cuban American, and Puerto Rican adolescents and young adults:findings from the Hispanic HANES. American Journal of Public Health; 1989.[135]<br>3.Booth M, Castro F, Anglin M. What do we know about Hispanic substance abuse? a review of the literature. In: Glick R, Moore J, editors. Drug use in Hispanic communities. New Brunswick, NJ: Rutgers University Press; 1989.[136]<br>4.Caetano R, Martinez R. Alcohol use in Madrid and among U.S. Hispanics. Berkeley, CA: The National Institute on Alcohol Abuse and Alcoholism; 1987 Contract No.: Report.[137]

A systematic review of school-based alcohol and other drug prevention programs facilitated by computers or the internet	Champion, K. E.;<br>Newton, N. C.;<br>Barrett, E. L.;<br>Teesson, M.	2011	12	1.Buller DB, Borland R, Woodall WG, Hall JR, Hines JM, Burris-Woodall P, et al. Randomized trials on consider this, a tailored, internet-delivered smoking prevention program for adolescents. Health education & behavior : the official publication of the Society for Public Health Education; 2008 Apr;35(2):260-81. PMID: 17114331. doi: 10.1177/1090198106288982.[138]<br>2.Norman CD, Maley O, Li X, Skinner HA. Using the internet to assist smoking prevention and cessation in schools: a randomized, controlled trial. Health Psychol; 2008 Nov;27(6):799-810. PMID: 19025276. doi: 10.1037/a0013105.[139]<br>3.Prokhorov AV, Kelder SH, Shegog R, Murray N, Peters R, Jr., Agurcia-Parker C, et al. Impact of A Smoking Prevention Interactive Experience (ASPIRE), an interactive, multimedia smoking prevention and cessation curriculum for culturally diverse high-school students. Nicotine & tobacco research : official journal of the Society for Research on Nicotine and Tobacco; 2008 Sep;10(9):1477-85. PMID: 19023839. doi: 10.1080/14622200802323183.[140]<br>4.Vogl L, Teesson M, Andrews G, Bird K, Steadman B, Dillon P. A computerized harm minimization prevention program for alcohol misuse and related harms: randomized controlled trial. Addiction (Abingdon, England); 2009 Apr;104(4):564-75. PMID: 19335655. doi: 10.1111/j.1360-0443.2009.02510.x.[141]<br>5.Newton NC, Vogl LE, Teesson M, Andrews G. CLIMATE Schools: alcohol module: cross-validation of a school-based prevention programme for alcohol misuse. Aust N Z J Psychiatry; 2009 Mar;43(3):201-7. PMID: 19221908. doi: 10.1080/00048670802653364.[142]<br>6.Newton NC, Andrews G, Teesson M, Vogl LE. Delivering prevention for alcohol and cannabis using the Internet: a cluster randomised controlled trial. Preventive medicine; 2009 Jun;48(6):579-84. PMID: 19389420. doi: 10.1016/j.ypmed.2009.04.009.[143]<br>7.Newton NC, Teesson M, Vogl LE, Andrews G. Internet-based prevention for alcohol and cannabis use: final results of the Climate Schools course. Addiction (Abingdon, England); 2010 Apr;105(4):749-59. PMID: 20148791. doi: 10.1111/j.1360-0443.2009.02853.x.[144]<br>8.Koning IM, Vollebergh WA, Smit F, Verdurmen JE, Van Den Eijnden RJ, Ter Bogt TF, et al. Preventing heavy alcohol use in adolescents (PAS): cluster randomized trial of a parent and student intervention offered separately and simultaneously. Addiction (Abingdon, England); 2009 Oct;104(10):1669-78. PMID: 21265908. doi: 10.1111/j.1360-0443.2009.02677.x.[145]<br>9.Aveyard P, Sherratt E, Almond J, Lawrence T, Lancashire R, Griffin C, et al. The change-in-stage and updated smoking status results from a cluster-randomized trial of smoking prevention and cessation using the transtheoretical model among British adolescents. Preventive medicine; 2001;33(4):313-24.[146]<br>10.Marsch L, Bickel W, Grabinski M. Application of interactive, computer technology to adolescent substance abuse prevention and treatment. Adolescent medicine: state of the art reviews; 2007;18(2):342-56, xii.[147]<br>11.Duncan TE, Duncan SC, Beauchamp N, Wells J, Ary DV. Development and evaluation of an interactive CD-ROM refusal skills program to prevent youth substance use: "refuse to use". Journal of behavioral medicine; 2000 Feb;23(1):59-72. PMID: 10749011.[148]<br>12.Lord SE, D'Amante D. 4: Efficacy of online alcohol and other drug prevention for early adolescents. Journal of Adolescent Health; 2007;40(2):S4.[149]

Computer-based programs for the prevention and management of illicit recreational drug use: a systematic review [19]	Wood, S. K.;<br>Eckley, L.;<br>Hughes, K.;<br>Hardcastle, K. A.;<br>Bellis, M. A.;<br>Schrooten, J.;<br>Demetrovics, Z.;<br>Voorham, L.	2014	12	1. Budney AJ, Fearer S, Walker DD, Stanger C, Thostenson J, Grabinski M, et al. An initial trial of a computerized behavioral intervention for cannabis use disorder. Drug and alcohol dependence; 2011 May 1;115(1-2):74-9. PMID: 21131143. doi: 10.1016/j.drugalcdep.2010.10.014.[150]<br>2. Deitz DK, Cook RF, Hendrickson A. Preventing prescription drug misuse: field test of the SmartRx Web program. Substance use & misuse; 2011;46(5):678-86. doi: 10.3109/10826084.2010.528124 [doi].[48]<br>3. Gilbert P, Ciccarone D, Gansky SA, Bangsberg DR, Clanon K, McPhee SJ, et al. Interactive "Video Doctor" counseling reduces drug and sexual risk behaviors among HIV-positive patients in diverse outpatient settings. PloS one; 2008 Apr 23;3(4):e1988. PMID: 18431475. doi: 10.1371/journal.pone.0001988.[151]<br>4. Kay-Lambkin FJ, Baker AL, Lewin TJ, Carr VJ. Computer-based psychological treatment for comorbid depression and problematic alcohol and/or cannabis use: a randomized controlled trial of clinical efficacy. Addiction (Abingdon, England); 2009 Mar;104(3):378-88. PMID: 19207345. doi: 10.1111/j.1360-0443.2008.02444.x.[152]<br>5. Lee CM, Neighbors C, Kilmer JR, Larimer ME. A brief, web-based personalized feedback selective intervention for college student marijuana use: a randomized clinical trial. Psychology of addictive behaviors: journal of the Society of Psychologists in Addictive Behaviors; 2010 Jun;24(2):265-73. PMID: 20565152. doi: 10.1037/a0018859.[153]<br>6. Marsch LA, Bickel WK, Badger GJ. Applying Computer Technology to Substance Abuse Prevention Science: Results of a Preliminary Examination. Journal of child & adolescent substance abuse; 2007 2007/03/06;16(2):69-94. doi: 10.1300/J029v16n02_04[73]<br>7. Newton NC, Teesson M, Vogl LE, Andrews G. Internet-based prevention for alcohol and cannabis use: final results of the Climate Schools course. Addiction (Abingdon, England); 2010 Apr;105(4):749-59. PMID: 20148791. doi: 10.1111/j.1360-0443.2009.02853.x.[144]<br>8. Schwinn TM, Schinke SP, Noia J. Preventing drug abuse among adolescent girls: outcome data from an internet-based intervention. Prevention science; 2010;11(1):24-32. doi: 10.1007/s11121-009-0146-9.[35]<br>9. Tossmann HP, Jonas B, Tensil MD, Lang P, Struber E. A controlled trial of an internet-based intervention program for cannabis users. Cyberpsychology, behavior and social networking; 2011 Nov;14(11):673-9. PMID: 21651419. doi: 10.1089/cyber.2010.0506.[154]<br>10. Williams C, Griffin KW, Macaulay AP, West TL, Gronewold E. Efficacy of a drug prevention CD-ROM intervention for adolescents. Substance use & misuse; 2005;40(6):869-78. PMID: 15974146.[155]
The effectiveness of electronic approaches to substance abuse prevention for adolescents [20]	Hopson, L.;<br>Wodarski, J.;<br>Tang, N.	2015	28	1. Aveyard P, Sherratt E, Almond J, Lawrence T, Lancashire R, Griffin C, et al. The change-in-stage and updated smoking status results from a cluster-randomized trial of smoking prevention and cessation using the transtheoretical model among British adolescents. Preventive medicine; 2001;33(4):313-24.[146]<br>2. Bersamin M, Paschall MJ, Fearnow-Kenney M, Wyrick D. Effectiveness of a Web-based alcohol-misuse and harm-prevention course among high- and low-risk students. Journal of American college health: J of ACH; 2007 Jan-Feb;55(4):247-54. PMID: 17319331. doi: 10.3200/jach.55.4.247-254.[156]<br>3. Bingham CR, Barretto AI, Walton MA, Bryant CM, Shope JT, Raghunathan TE. Efficacy of a web-based, tailored, alcohol prevention/intervention program for college students: 3-month follow-up. Journal of drug education; 2011;41(4):405-30. PMID: 22455103. doi: 10.2190/DE.41.4.e.[157]<br>4. Bishop D, Bryant KS, Giles SM, Hansen WB, Dusenbury L. Simplifying the delivery of a prevention program with web-based enhancements. The journal of primary prevention; 2006 Jul;27(4):433-44. PMID: 16763766. doi:

				10.1007/s10935-006-0042-z.Buller, D. B., Borland, R., Woodall, W. G., Hall, J. R., Hines, J. M., Burris-Woodall, P., ... Saba, L. (2008). [158]<br>5. Bersamin M, Paschall MJ, Fearnow-Kenney M, Wyrick D. Effectiveness of a Web-based alcohol-misuse and harm-prevention course among high- and low-risk students. Journal of American college health: J of ACH; 2007 Jan-Feb;55(4):247-54. PMID: 17319331. doi: 10.3200/jach.55.4.247-254. [138]<br>6. Croom K, Lewis D, Marchell T, Lesser ML, Reyna VF, Kubicki-Bedford L, et al. Impact of an online alcohol education course on behavior and harm for incoming first-year college students: short-term evaluation of a randomized trial. Journal of American college health: J of ACH; 2009 Jan-Feb;57(4):445-54. PMID: 19114384. doi: 10.3200/jach.57.4.445-454.[159]<br>7. Di Noia J, Schwinn TM, Dastur ZA, Schinke SP. The relative efficacy of pamphlets, CD-ROM, and the Internet for disseminating adolescent drug abuse prevention programs: an exploratory study. Preventive medicine; 2003 Dec;37(6 Pt 1):646-53. PMID: 14636798.[160]<br>8. Epstein J, Collins KK, Thomson NR, Pancella T, Pauley D. The doubles: evaluation of a substance abuse education curriculum for elementary school students. Journal of child & adolescent substance abuse; 2007;16(4):1-22.[161]<br>9. Hansen WB, Bishop DC, Bryant KS. Using online components to facilitate program implementation: impact of technological enhancements to all stars on ease and quality of program delivery. Prevention science: the official journal of the Society for Prevention Research; 2009 Mar;10(1):66-75. PMID: 19067165. doi: 10.1007/s11121-008-0118-5 [doi]. [57]<br>10. Hecht ML, Marsiglia FF, Elek E, Wagstaff DA, Kulis S, Dustman P, et al. Culturally grounded substance use prevention: an evaluation of the keepin' it R.E.A.L. curriculum. Prevention science: the official journal of the Society for Prevention Research; 2003 Dec;4(4):233-48. PMID: 14598996.[162]<br>11. Hustad JT, Barnett NP, Borsari B, Jackson KM. Web-based alcohol prevention for incoming college students: a randomized controlled trial. Addictive behaviors; 2010 Mar;35(3):183-9. PMID: 19900763. doi: 10.1016/j.addbeh.2009.10.012.[163]<br>12. Koning IM, van den Eijnden RJ, Verdurmen JE, Engels RC, Vollebergh WA. Long-term effects of a parent and student intervention on alcohol use in adolescents: a cluster randomized controlled trial. American journal of preventive medicine; 2011 May;40(5):541-7. PMID: 21496753. doi: 10.1016/j.amepre.2010.12.030.[164]<br>13. Kypri K, Saunders JB, Williams SM, McGee RO, Langley JD, Cashell-Smith ML, et al. Web-based screening and brief intervention for hazardous drinking: a double-blind randomized controlled trial. Addiction (Abingdon, England); 2004 Nov;99(11):1410-7. PMID: 15500594. doi: 10.1111/j.1360-0443.2004.00847.x.[165]<br>14. Lord SE, D'Amante D. 4: Efficacy of online alcohol and other drug prevention for early adolescents. Journal of Adolescent Health; 2007;40(2):S4. [149]<br>15. Moore MJ, Soderquist J, Werch C. Feasibility and efficacy of a binge drinking prevention intervention for college students delivered via the Internet versus postal mail. Journal of American college health: J of ACH; 2005 Jul-Aug;54(1):38-44. PMID: 16050327. doi: 10.3200/jach.54.1.38-44.[166]<br>16. Neighbors C, Lewis MA, Atkins DC, Jensen MM, Walter T, Fossos N, et al. Efficacy of web-based personalized normative feedback: a two-year randomized controlled trial. Journal of consulting and clinical psychology; 2010 Dec;78(6):898-911. PMID: 20873892. doi: 10.1037/a0020766.[167]<br>17. Newton NC, Teesson M, Vogl LE, Andrews G. Internet-based prevention for alcohol and cannabis use: final results of the Climate Schools course. Addiction (Abingdon, England); 2010 Apr;105(4):749-59. PMID: 20148791. doi: 10.1111/j.1360-0443.2009.02853.x. [142]

				18. Norman CD, Maley O, Li X, Skinner HA. Using the internet to assist smoking prevention and cessation in schools: a randomized, controlled trial. Health Psychol; 2008 Nov;27(6):799-810. PMID: 19025276. doi: 10.1037/a0013105.[139]<br>19. Prokhorov AV, Kelder SH, Shegog R, Murray N, Peters R, Jr., Agurcia-Parker C, et al. Impact of A Smoking Prevention Interactive Experience (ASPIRE), an interactive, multimedia smoking prevention and cessation curriculum for culturally diverse high-school students. Nicotine & tobacco research: official journal of the Society for Research on Nicotine and Tobacco; 2008 Sep;10(9):1477-85. PMID: 19023839. doi: 10.1080/14622200802323183.[140]<br>20. Rohrbach LA, Gunning M, Sun P, Sussman S. The Project towards No Drug Abuse (TND) dissemination trial: implementation fidelity and immediate outcomes. Prevention science: the official journal of the Society for Prevention Research; 2010 Mar;11(1):77-88. PMID: 19757052. doi: 10.1007/s11121-009-0151-z.[168]<br>21. Schinke SP, Fang L, Cole KC, Cohen-Cutler S. Preventing substance use among Black and Hispanic adolescent girls: results from a computer-delivered, mother-daughter intervention approach. Substance use & misuse; 2011;46(1):35-45. doi: 10.3109/10826084.2011.521074 [doi].[52]<br>22. Schinke SP, Cole KC, Fang L. Gender-specific intervention to reduce underage drinking among early adolescent girls: a test of a computer-mediated, mother-daughter program. Journal of studies on alcohol and drugs; 2009 Jan;70(1):70-7. PMID: 19118394.[169]<br>23. Schinke SP, Schwinn TM, Di Noia J, Cole KC. Reducing the risks of alcohol use among urban youth: three-year effects of a computer-based intervention with and without parent involvement. J Stud Alcohol; 2004 Jul;65(4):443-9. PMID: 15376818.[170]<br>24. Schwinn TM, Schinke SP, Noia J. Preventing drug abuse among adolescent girls: outcome data from an internet-based intervention. Prevention science; 2010;11(1):24-32. doi: 10.1007/s11121-009-0146-9. [35]<br>25. Vogl L, Teesson M, Andrews G, Bird K, Steadman B, Dillon P. A computerized harm minimization prevention program for alcohol misuse and related harms: randomized controlled trial. Addiction (Abingdon, England); 2009 Apr;104(4):564-75. PMID: 19335655. doi: 10.1111/j.1360-0443.2009.02510.x. [141]<br>26. Walters ST, Vader AM, Harris TR. A controlled trial of web-based feedback for heavy drinking college students. Prevention science: the official journal of the Society for Prevention Research; 2007 Mar;8(1):83-8. PMID: 17136461. doi: 10.1007/s11121-006-0059-9.[171]<br>27. Warren JR, Hecht ML, Wagstaff DA, Elek E, Ndiaye K, Dustman P, et al. Communicating prevention: The effects of the keepin'it REAL classroom videotapes and televised PSAs on middle-school students' substance use. Journal of Applied Communication Research; 2006;34(2):209-27.[172]<br>28. Williams C, Griffin KW, Macaulay AP, West TL, Gronewold E. Efficacy of a drug prevention CD-ROM intervention for adolescents. Substance use & misuse; 2005;40(6):869-78. PMID: 15974146. [155]

Prevention of alcohol and other drug use and related harm in the digital age: what does the evidence tell us? [26]	Champion, K. E.;<br>Newton, N. C.;<br>Teesson, M.	2016	12	1. de Josselin de Jong S, Candel M, Segaar D, Cremers HP, de Vries H. Efficacy of a Web-based computer-tailored smoking prevention intervention for Dutch adolescents: randomized controlled trial. Journal of medical Internet research; 2014 Mar 21;16(3):e82. PMID: 24657434. doi: 10.2196/jmir.2469.[173]<br>2. Doumas DM, Esp S, Turrisi R, Hausheer R, Cuffee C. A test of the efficacy of a brief, web-based personalized feedback intervention to reduce drinking among 9th grade students. Addictive behaviors; 2014 Jan;39(1):231-8. PMID: 24148137. doi: 10.1016/j.addbeh.2013.10.011.[174]<br>3. Doumas DM, Hausheer R, Esp S, Cuffee C. Reducing alcohol use among 9th grade students: 6 month outcomes of a brief, Web-based intervention. Journal of substance abuse treatment; 2014 Jul;47(1):102-5. PMID: 24666810. doi: 10.1016/j.jsat.2014.02.006.[175]<br>4. Vogl LE, Newton NC, Champion KE, Teesson M. A universal harm-minimisation approach to preventing psychostimulant and cannabis use in adolescents: a cluster randomised controlled trial. Substance abuse treatment, prevention, and policy; 2014 Jun 18;9:24. PMID: 24943829. doi: 10.1186/1747-597x-9-24.[176]<br>5. Champion KE, Newton NC, Stapinski L, Slade T, Barrett EL, Teesson M. A cross-validation trial of an Internet-based prevention program for alcohol and cannabis: Preliminary results from a cluster randomised controlled trial. The Australian and New Zealand Journal of Psychiatry; 2016;50(1):64-73. doi: 10.1177/0004867415577435 [doi]. [40]<br>6. Newton NC, Andrews G, Champion KE, Teesson M. Universal Internet-based prevention for alcohol and cannabis use reduces truancy, psychological distress and moral disengagement: a cluster randomised controlled trial. Preventive medicine; 2014;65:109-15. doi: 10.1016/j.ypmed.2014.05.003 [doi]. [39]<br>7. Malmberg M, Kleinjan M, Overbeek G, Vermulst A, Lammers J, Monshouwer K, et al. Substance use outcomes in the Healthy School and Drugs program: results from a latent growth curve approach. Addictive behaviors; 2015 Mar;42:194-202. PMID: 25481454. doi: 10.1016/j.addbeh.2014.11.021.[177]<br>8. Malmberg M, Kleinjan M, Overbeek G, Vermulst A, Monshouwer K, Lammers J, et al. Effectiveness of the 'Healthy School and Drugs' prevention programme on adolescents' substance use: a randomized clustered trial. Addiction (Abingdon, England); 2014 Jun;109(6):1031-40. PMID: 24612164. doi: 10.1111/add.12526.[178]<br>9. Velicer WF, Redding CA, Paiva AL, Mauriello LM, Blissmer B, Oatley K, et al. Multiple behavior interventions to prevent substance abuse and increase energy balance behaviors in middle school students. Transl Behav Med; 2013 Mar;3(1):82-93. PMID: 23585821. doi: 10.1007/s13142-013-0197-0.[179]<br>10. Bannink R, Broeren S, Joosten-van Zwanenburg E, van As E, van de Looij-Jansen P, Raat H. Effectiveness of a Web-based tailored intervention (E-health4Uth) and consultation to promote adolescents' health: randomized controlled trial. Journal of medical Internet research; 2014 May 30;16(5):e143. PMID: 24878521. doi: 10.2196/jmir.3163.[180]<br>11. Rundle-Thiele S, Schuster L, Dietrich T, Russell-Bennett R, Drennan J, Leo C, et al. Maintaining or changing a drinking behavior? GOKA's short-term outcomes. Journal of Business Research; 2015;68(10):2155-63.[181]<br>12. Walton MA, Resko S, Barry KL, Chermack ST, Zucker RA, Zimmerman MA, et al. A randomized controlled trial testing the efficacy of a brief cannabis universal prevention program among adolescents in primary care. Addiction (Abingdon, England); 2014 May;109(5):786-97. PMID: 24372937. doi: 10.1111/add.12469.[182]

Mass media interventions for preventing smoking in young people [29]	Carson, K. V.;<br>Ameer, F.;<br>Sayehmiri, K;<br>Hnin, K.;<br>Vanagteren, J. E.;<br>Sayehmiri, F.;<br>Brinn, M. P.;<br>Esterman, A. J.;<br>Chang, A. B.;<br>Smith, B. J.	2017; 2010	8; 7	1. Bauman 1991<br>a. Bauman KE, LaPrelle J, Brown JD, Koch GG, Padgett CA. The influence of three mass media campaigns on variables related to adolescent cigarette smoking: results of a field experiment. American journal of public health; 1991 May;81(5):597-604. PMID: 2014859.[183]<br>b. Bauman KE, Brown JD, Bryan ES, Fisher LA, Padgett CA, Sweeney JM. Three mass media campaigns to prevent adolescent cigarette smoking. Preventive medicine; 1988 Sep;17(5):510-30. PMID: 3237655.[184]<br>c. Bauman KE, Padgett CA, Koch GG. A media-based campaign to encourage personal communication among adolescents about not smoking cigarettes: participation, selection and consequences. Health education research; 1989;4(1):35-44. doi: 10.1093/her/4.1.35.[185]<br>d. Brown JD, Bauman KE, Padgett CA. A validity problem in measuring exposure to mass media campaigns. Health Educ Q; 1990 Fall;17(3):299-306. PMID: 2228632.[186]<br>e. L J, Bauman KE, Koch GG. High Intercommunity Variation in Adolescent Cigarette Smoking in a 10-Community Field Experiment. Evaluation Review; 1992;16(2):115-30. doi: 10.1177/0193841x9201600201Fallin A, Neilands TB, Jordan JW, Hong JS, Ling PM. [187]<br>2. Fallin A, Neilands TB, Jordan JW, Hong JS, Ling PM. Wreaking "havoc" on smoking: social branding to reach young adult "partiers" in Oklahoma. American journal of preventive medicine; 2015 Jan;48(1 Suppl 1):S78-85. PMID: 25528713. doi: 10.1016/j.amepre.2014.09.008.[188]<br>3. Flay 1995<br>f. Flay BR, Miller TQ, Hedeker D, Siddiqui O, Britton CF, Brannon BR, et al. The television, school, and family smoking prevention and cessation project. VIII. Student outcomes and mediating variables. Preventive medicine; 1995 Jan;24(1):29-40. PMID: 7740012. doi: 10.1006/pmed.1995.1005.[181]<br>g. Brannon BR, Dent CW, Flay BR, Smith G, Sussman S, Pentz MA, et al. The television, school, and family project. V. The impact of curriculum delivery format on program acceptance. Preventive medicine; 1989;18(4):492-502. [51]<br>h. Flay BR, Brannon BR, Johnson CA, Hansen WB, Ulene AL, Whitney-Saltiel DA, et al. The television school and family smoking prevention and cessation project. 1. Theoretical basis and program development. Preventive medicine; 1988 Sep;17(5):585-607. PMID: 3237658.[190]<br>i. Sussman S, Brannon BR, Flay BR, Gleason L, Senor S, Sobol DF, et al. The television, school and family smoking prevention/cessation project. II. Formative evaluation of television segments by teenagers and parents – implications for parental involvement in drug education. Health education research; 1986;1(3):185-94. doi: 10.1093/her/1.3.185.[191]<br>j. Sussman S, Dent CW, Brannon BR, Glowacz K, Gleason LR, Ullery S, et al. The television, school and family smoking prevention/cessation project. IV. Controlling for program success expectancies across experimental and control conditions. Addictive behaviors; 1989;14(6):601-10. PMID: 2618843[192]<br>4. Flynn 1995<br>k. Flynn BS, Worden JK, Secker-Walker RH, Badger GJ, Geller BM. Cigarette Smoking Prevention Effects of Mass Media and School Interventions Targeted to Gender and Age Groups. Journal of Health Education; 1995 1995/04/01;26(sup2):S45-S51. doi: 10.1080/10556699.1995.10603147.[193]<br>l. Flynn BS, Worden JK, Secker-Walker RH, Badger GJ, Geller BM, Costanza MC. Prevention of cigarette smoking through mass media intervention and school programs. American journal of public health; 1992 Jun;82(6):827-34. PMID: 1585963.[194]<br>m. Flynn BS, Worden JK, Secker-Walker RH, Pirie PL, Badger GJ, Carpenter JH. Long-term responses of higher and lower risk youths to smoking prevention interventions. Preventive medicine; 1997 May-Jun;26(3):389-94. PMID: 9144764. doi: 10.1006/pmed.1997.0159.[195]<br>n. Flynn BS, Worden JK, Secker-Walker RH, Pirie PL, Badger GJ,

Beyond face-to-face individual counseling: A systematic review on alternative modes of motivational interviewing in substance abuse treatment and prevention [50]	Jiang, S.;<br>Wu, L.;<br>Gao, X.	2017	25	1. Stotts AL, Diclemente CC, Dolan-Mullen P. One-to-one: a motivational intervention for resistant pregnant smokers. Addictive behaviors; 2002 Mar-Apr;27(2):275-92. PMID: 11817768.[212]<br>2. Rigotti NA, Park ER, Regan S, Chang Y, Perry K, Loudin B, et al. Efficacy of telephone counseling for pregnant smokers: a randomized controlled trial. Obstet Gynecol; 2006 Jul;108(1):83-92. PMID: 16816060. doi: 10.1097/01.AOG.0000218100.05601.f8.[213]<br>3. Peterson AV, Jr., Kealey KA, Mann SL, Marek PM, Ludman EJ, Liu J, et al. Group-randomized trial of a proactive, personalized telephone counseling intervention for adolescent smoking cessation. Journal of the National Cancer Institute; 2009 Oct 21;101(20):1378-92. PMID: 19822836. doi: 10.1093/jnci/djp317.[214]<br>4. Severson HH, Peterson AL, Andrews JA, Gordon JS, Cigrang JA, Danaher BG, et al. Smokeless tobacco cessation in military personnel: a randomized controlled trial. Nicotine & tobacco research: official journal of the Society for Research on Nicotine and Tobacco; 2009 Jun;11(6):730-8. PMID: 19395686. doi: 10.1093/ntr/ntp057.[215]<br>5. Bastian LA, Fish LJ, Peterson BL, Biddle AK, Garst J, Lyna P, et al. Assessment of the impact of adjunctive proactive telephone counseling to promote smoking cessation among lung cancer patients' social networks. American journal of health promotion: AJHP; 2013 Jan-Feb;27(3):181-90. PMID: 23286595. doi: 10.4278/ajhp.101122-QUAN-387.[216]<br>6. Jimenez-Muro A, Nerin I, Samper P, Marqueta A, Beamonte A, Gargallo P, et al. A proactive smoking cessation intervention in postpartum women. Midwifery; 2013 Mar;29(3):240-5. PMID: 22361008. doi: 10.1016/j.midw.2012.01.003.[217]<br>7. Woodruff SI, Conway TL, Edwards CC, Elliott SP, Crittenden J. Evaluation of an Internet virtual world chat room for adolescent smoking cessation. Addictive behaviors; 2007 Sep;32(9):1769-86. PMID: 17250972. doi: 10.1016/j.addbeh.2006.12.008.[218]<br>8. Norman CD, Maley O, Li X, Skinner HA. Using the internet to assist smoking prevention and cessation in schools: a randomized, controlled trial. Health Psychol; 2008 Nov;27(6):799-810. PMID: 19025276. doi: 10.1037/a0013105. [139]<br>9. Becker J, Haug S, Sullivan R, Schaub MP. Effectiveness of different Web-based interventions to prepare co-smokers of cigarettes and cannabis for double cessation: a three-arm randomized controlled trial. Journal of medical Internet research; 2014 Dec 5;16(12):e273. PMID: 25486674. doi: 10.2196/jmir.3246.[219]<br>10. Christoff Ade O, Boerngen-Lacerda R. Reducing substance involvement in college students: a three-arm parallel-group randomized controlled trial of a computer-based intervention. Addictive behaviors; 2015;45:164-71. doi: 10.1016/j.addbeh.2015.01.019 [doi]. [42]<br>11. Mason MJ, Campbell L, Way T, Keyser-Marcus L, Benotsch E, Mennis J, et al. Development and Outcomes of a Text Messaging Tobacco Cessation Intervention With Urban Adolescents. Substance abuse; 2015;36(4):500-6. PMID: 25551337. doi: 10.1080/08897077.2014.987946.[220]<br>12. Blankers M, Koeter MW, Schippers GM. Internet therapy versus internet self-help versus no treatment for problematic alcohol use: A randomized controlled trial. Journal of consulting and clinical psychology; 2011 Jun;79(3):330-41. PMID: 21534652. doi: 10.1037/a0023498.[221]<br>13. Brown RL, Saunders LA, Bobula JA, Mundt MP, Koch PE. Randomized-controlled trial of a telephone and mail intervention for alcohol use disorders: three-month drinking outcomes. Alcoholism, clinical and experimental research; 2007 Aug;31(8):1372-9. PMID: 17550366. doi: 10.1111/j.1530-0277.2007.00430.x.[222]<br>14. Borsari B, Short EE, Mastroleo NR, Hustad JT, Tevyaw TO, Barnett NP, et al. Phone-delivered brief motivational interventions for mandated college students delivered during the summer months. Journal of substance abuse treatment; 2014 May-Jun;46(5):592-6. PMID: 24512944. doi: 10.1016/j.jsat.2014.01.001.[223]<br>15. LaChance H, Feldstein Ewing SW, Bryan AD, Hutchison KE. What makes group MET work? A randomized controlled trial of college student drinkers in mandated alcohol diversion. Psychology of addictive behaviors : journal of the Society of Psychologists in Addictive Behaviors; 2009 Dec;23(4):598-612. PMID: 20025366. doi: 10.1037/a0016633.[224]<br>16. Wongpakaran T, Petcharaj K, Wongpakaran N, Sombatmai S, Boriputtakul T, Intarakamhaeng D, et al. The effect of telephone-based intervention (TBI) in alcohol abusers: a pilot study. Journal of the Medical

A Systematic Review of the mHealth Interventions to Prevent Alcohol and Substance Abuse [59]	Kazemi, D. M.;<br>Borsari, B.;<br>Levine, M. J.;<br>Li, S.;<br>Lamberson, K. A.;<br>Matta, L. A.	2017	12	1. Agyapong VI, Ahern S, McLoughlin DM, Farren CK. Supportive text messaging for depression and comorbid alcohol use disorder: single-blind randomised trial. Journal of affective disorders; 2012 Dec 10;141(2-3):168-76. PMID: 22464008. doi: 10.1016/j.jad.2012.02.040.[235]<br>2. Weitzel JA, Bernhardt JM, Usdan S, Mays D, Glanz K. Using wireless handheld computers and tailored text messaging to reduce negative consequences of drinking alcohol. Journal of studies on alcohol and drugs; 2007 Jul;68(4):534-7. PMID: 17568957.[236]<br>3. Suffoletto B, Callaway C, Kristan J, Kraemer K, Clark DB. Text-message-based drinking assessments and brief interventions for young adults discharged from the emergency department. Alcoholism, clinical and experimental research; 2012 Mar;36(3):552-60. PMID: 22168137. doi: 10.1111/j.1530-0277.2011.01646.x.[237]<br>4. Haug S, Schaub MP, Venzin V, Meyer C, John U, Gmel G. A pre-post study on the appropriateness and effectiveness of a Web- and text messaging-based intervention to reduce problem drinking in emerging adults. Journal of medical Internet research; 2013 Sep 2;15(9):e196. PMID: 23999406. doi: 10.2196/jmir.2755.[238]<br>5. Suffoletto B, Kristan J, Callaway C, Kim KH, Chung T, Monti PM, et al. A text message alcohol intervention for young adult emergency department patients: a randomized clinical trial. Annals of emergency medicine; 2014 Dec;64(6):664-72.e4. PMID: 25017822. doi: 10.1016/j.annemergmed.2014.06.010.[230]<br>6. Gajecki M, Berman AH, Sinadinovic K, Rosendahl I, Andersson C. Mobile phone brief intervention applications for risky alcohol use among university students: a randomized controlled study. Addiction science & clinical practice; 2014 Jul 2;9:11. PMID: 24985342. doi: 10.1186/1940-0640-9-11.[239]<br>7. Gonzales R, Ang A, Murphy DA, Glik DC, Anglin MD. Substance use recovery outcomes among a cohort of youth participating in a mobile-based texting aftercare pilot program. Journal of substance abuse treatment; 2014 Jul;47(1):20-6. PMID: 24629885. doi: 10.1016/j.jsat.2014.01.010.[240]<br>8. Gustafson DH, McTavish FM, Chih MY, Atwood AK, Johnson RA, Boyle MG, et al. A smartphone application to support recovery from alcoholism: a randomized clinical trial. JAMA psychiatry; 2014 May;71(5):566-72. PMID: 24671165. doi: 10.1001/jamapsychiatry.2013.4642.[241]<br>9. Lucht MJ, Hoffman L, Haug S, Meyer C, Pussehl D, Quellmalz A, et al. A surveillance tool using mobile phone short message service to reduce alcohol consumption among alcohol-dependent patients. Alcoholism, clinical and experimental research; 2014 Jun;38(6):1728-36. PMID: 24730528. doi: 10.1111/acer.12403.[242]<br>10. Shrier LA, Rhoads A, Burke P, Walls C, Blood EA. Real-time, contextual intervention using mobile technology to reduce marijuana use among youth: a pilot study. Addictive behaviors; 2014 Jan;39(1):173-80. PMID: 24139665. doi: 10.1016/j.addbeh.2013.09.028.[243]<br>11. Haug S, Lucht MJ, John U, Meyer C, Schaub MP. A pilot study on the feasibility and acceptability of a text message-based aftercare treatment programme among alcohol outpatients. Alcohol and alcoholism (Oxford, Oxfordshire); 2015 Mar;50(2):188-94. PMID: 25600249. doi: 10.1093/alcalc/agu107.[244]<br>12. Gonzalez VM, Dulin PL. Comparison of a smartphone app for alcohol use disorders with an Internet-based intervention plus bibliotherapy: A pilot study. Journal of consulting and clinical psychology; 2015 Apr;83(2):335-45. PMID: 25622202. doi: 10.1037/a0038620.[245]
